# Microwave Technique for Linear Skull Fracture Detection—Simulation and Experimental Study Using Realistic Human Head Models

**DOI:** 10.3390/bios14090434

**Published:** 2024-09-06

**Authors:** Mariella Särestöniemi, Daljeet Singh, Mikael von und zu Fraunberg, Teemu Myllylä

**Affiliations:** 1 Research Unit of Health Sciences and Technology, Faculty of Medicine, University of Oulu, 90014 Oulu, Finland; daljeet.singh@oulu.fi (D.S.); teemu.myllyla@oulu.fi (T.M.); 2 Centre for Wireless Communications, University of Oulu, 90014 Oulu, Finland; 3 InfoTechOulu, Oulu University, 90014 Oulu, Finland; 4 Oulu University Hospital, 90240 Oulu, Finland; mikael.fraunberg@ppshp.fi; 5 Medical Research Center (MRC) Oulu, 90240 Oulu, Finland; 6 Optoelectronics and Measurement Techniques Research Unit, University of Oulu, 90014 Oulu, Finland

**Keywords:** body area networks, bone fracture, bone healing process, emerging medical applications, in-body propagation, skull fractures, tissue phantoms, ultrawideband body area networks, phantoms

## Abstract

Microwave (MW) sensing is regarded as a promising technique for various medical monitoring and diagnostic applications due to its numerous advantages and the potential to be developed into a portable device for use outside hospital settings. The detection of skull fractures and the monitoring of their healing process would greatly benefit from a rapidly and frequently usable application that can be employed outside the hospital. This paper presents a simulation- and experiment-based study on skull fracture detection with the MW technique using realistic models for the first time. It also presents assessments on the most promising frequency ranges for skull fracture detection within the Industrial, Scientific and Medical (ISM) and ultrawideband (UWB) ranges. Evaluations are carried out with electromagnetic simulations using different head tissue layer models corresponding to different locations in the human head, as well as an anatomically realistic human head simulation model. The measurements are conducted with a real human skull combined with tissue phantoms developed in our laboratory. The comprehensive evaluations show that fractures cause clear differences in antenna and channel parameters (S11 and S21). The difference in S11 is 0.1–20 dB and in S21 is 0.1–30 dB, depending on the fracture width and location. Skull fractures with a less than 1 mm width can be detected with microwaves at different fracture locations. The detectability is frequency dependent. Power flow representations illustrate how fractures impact on the signal propagation at different frequencies. MW-based detection of skull fractures provides the possibility to (1) detect fractures using a safe and low-cost portable device, (2) monitor the healing-process of fractures, and (3) bring essential information for emerging portable MW-based diagnostic applications that can detect, e.g., strokes.

## 1. Introduction

Skull fractures typically result from significant cranial impacts, such as those sustained during falls, vehicular accidents, or sports-related injuries. Various types of skull fractures exist, including linear, depressed, basial, diastatic, and compound fractures, each characterized by distinct features and clinical implications [1].

The early and rapid diagnosis of skull fractures offers several advantages, including the prevention of complications and improved treatment outcomes. Thus, the early-phase determination of a skull fracture’s severity, treatment assessment, and hospitalization requirements is critical. Recent advancements, such as high-resolution CT scans and MRI, have significantly enhanced the detection and evaluation of skull fractures, facilitating more precise and timely medical interventions. For skull fractures, the CT scan has been the best option so far since it can diagnose fractures and important related injuries, such as brain hemorrhage. However, these exiting techniques have limitations in terms of radiation exposure and accessibility. A comprehensive literature survey of skull fracture detection methods can be found in [2].

Recent interest has emerged in novel, safer techniques that could enable the detection and monitoring of skull fractures, including portable applications. For instance, point-of-care ultrasound (POCUS) has been considered as a good alternative for skull fracture detection, especially in children [3,4]. It is non-invasive, non-ionizing, and cost-effective method to detect skull fractures quickly and with a portable device. However, the main challenge with POCUS is that its accuracy heavily relies on the skill and experience of the operator. Inexperienced users may miss fractures or misinterpret findings [3].

Emerging methods for bone monitoring include microwave (MW) and near-infrared spectroscopy (NIRS) techniques. Both methods offer non-ionizing, safe ways to monitor bone health with fast measurement procedures and good spatial resolution. Additionally, these techniques can be implemented in small, portable devices, making them usable outside hospitals, such as in ambulances.

The NIRS-based bone health monitoring technique has only recently been initiated [5], whereas the MW technique was proposed for monitoring bone mineral density as early as 2015 [6]. This study was further extended to monitor the progression of osteoporosis using the UWB radar technique in a distal femur model [7]. In 2016, a frequency domain analysis of hip fractures using microwaves was presented in [8]. This study utilized a split ring resonator sensor and phantom models. It was later extended to evaluate the MW technique for monitoring the healing process of leg fractures using a numerical study [9]. The study provided promising results for real-time monitoring of bone healing and inspired further research on leg bone fracture detection [10,11,12]. These studies showed promising results in accurately detecting even small-sized fractures.

The microwave technique has also been applied to monitor the healing process of skull fractures after surgery in several studies. In [13], microwaves were proposed for the first time to monitor skull healing after craniotomy. The simulation-based study was conducted using a layered head model with a transmission line and calculating the reflected pulse at the interface between layers. Skull healing was monitored by analyzing shifts in the time domain corresponding to variations of skull thickness.

The microwave-based skull fracture healing monitoring study was continued in [14] using a UWB sensor based on a resistively loaded dipole antenna and cranial surgery phantom models. The evaluations demonstrated that the progression of skull healing could be monitored by analyzing variations in the amplitude of the reflected pulses corresponding to different healing stages. While the results were promising for monitoring the post-surgery healing process, they are not directly applicable for detecting skull fractures due to injury. A microwave sensor utilizing a split ring resonator has been studied both in the lab and clinically to aid bone healing in pediatric patients with craniosynostosis, a condition where the skull sutures fuse prematurely [15,16,17]. The previously published studies presented extensive simulations and measurement results, as well as clinical validations for monitoring the healing process after the cranial surgery using certain types of microwave sensors. However, none of the studies present results for detecting linear skull fractures due to the injury nor presented healing process results with such linear fractures. Additionally, previous results do not present channel parameter evaluations in fracture monitoring, which, in many cases, provides better detectability than only analyzing antenna impedance. Moreover, the previously presented literature has not assessed most promising frequencies for skull fracture detection by presenting evaluation results for wide frequency bands and using different antenna types.

In general, the MW technique is considered a promising method for detecting various tissue abnormalities, particularly for portable monitoring applications and point-of-care diagnostics [18,19]. The basic principle behind detecting abnormalities in human tissues using microwaves is that the dielectric properties of abnormalities differ from those of the surrounding tissue [20,21]. These differences cause changes in signal propagation between antennas located near the abnormality. Abnormalities can be detected by comparing antenna and channel parameter data measured from the patient to reference data sets generated for healthy cases. The measured channel parameters can be used to produce images using MW imaging algorithms; however, it can also be based on a pure channel parameter analysis to provide information on the presence of abnormalities without images [22]. Microwave-based detection of brain abnormalities has been explored, particularly in the context of stroke detection [23]. For hemorrhagic strokes, identifying initial skull fractures is crucial, as they can significantly impact channel analysis-based stroke detection. Skull fractures also alter the measured channel responses. In cases of traumatic brain injuries, which often involve head concussions, detecting potential skull bone fractures is of high importance.

This paper presents for the first time a realistic simulation- and experiment-based study on skull fracture detection with the MW technique using several different head models, including 3D realistic models. Compared to the other MW-based bone fracture detection studies, the novelty of this study is the use of realistic models to validate comprehensively the usability of MW sensing in the detection of linear fractures of different sizes and how fractures affect antenna and channel parameters. Additionally, this paper presents for the first time how the detectability of skull fractures is frequency dependent by showing the varying impact of the skull fractures in the frequency range of 2–10 GHz, which covers the Industrial, Scientific and Medical (ISM) and ultrawideband (UWB) ranges. This study also provides assessments for the most optimal frequency ranges for the skull fracture detection. The study is carried out with electromagnetic simulations and experiments using a real human skull and tissue-mimicking phantoms. The simulations are conducted with head tissue layer models resembling different locations in the head, as well as with an anatomical human head model. The evaluations are carried out using a small flexible antenna designed for contact sensing and a directional cavity-backed antenna designed for non-contact sensing. Linear fracture types with different sizes are evaluated. Additionally, the possibility of monitoring the fracture healing process is assessed.

## 2. Materials and Methods

### 2.1. Bone Fracture Detection with Microwaves

Bone fractures are conventionally modelled with the blood by which fractures are filled in an acute injury [1]. In the healing process, fractures should be modelled as connective tissue [24]. The core concept of detecting bone fractures using microwaves hinges on the clear difference in dielectric properties between blood/connective tissue and bone tissue. This contrast is illustrated in Table 1, which details the average dielectric properties, i.e., relative permittivity and conductivity, of human tissues based on Gabriel’s dispersion relationships [21].

In general, when a radio signal encounters the boundary between two tissues/materials with different dielectric properties, several phenomenon can occur: (a) reflection, in which part of the signal is reflected back into the original tissue, (b) scattering in various directions due to the irregulates at the boundaries, and (c) transmission, in which some of the signal components continue to propagate into the second tissue but their direction and strength may be altered. These signal components may sum positively or negatively in the receiving antenna. Other factors influencing signal propagation through tissues include the antenna’s radiation characteristics, such as its frequency, polarization, and radiation pattern, as well as the antenna’s placement on the skin. All these factors cause changes in signal propagation in the vicinity of the bone fracture and, hence, bone fractures can be detected, e.g., from the antenna reflection coefficient S11 and channel parameters S21, S31, and S*N*1, in which *N* is the number of antennas. Additionally, fractures can be analyzed in time domain responses, as shown in [7].

Frequency selection plays a crucial role in bone fracture detection due to three reasons. Firstly, the difference in dielectric properties of the bone and blood/connective tissue vary clearly at different frequencies and, hence, the differences in S11 and S21 could be more visible in the frequencies where differences are largest. Secondly, the wavelength in the tissue influences the detection accuracy. Conventionally, the higher the frequency, the smaller the wavelength, and hence the better the fracture detection resolution [25]. Additionally, the radiation characteristics of the antenna can have a significant impact on detection. Due to these several aspects, optimal frequency selection for skull fracture monitoring is not straightforward and thus requires comprehensive investigations.

Table 1 delineates the dielectric properties of human head tissue pertinent to this study at frequencies of 2, 4, 6, 8, and 10 GHz. Additionally, the dielectric properties of blood and connective tissue, which are commonly utilized in bone fracture modeling during acute injury and healing phases, are included. The distinctions between these tissues and bone tissue are also highlighted. Furthermore, the wavelength values within these tissues are provided to elucidate the detection accuracy across different frequencies. When comparing dielectric properties of bones, blood, and connective tissues, it is noted that differences are clear, especially in relative permittivity values, both with blood as well as with connective tissues. Additionally, there are clear differences in conductivity values, which increase remarkably with frequency. Wavelengths in bone tissues are small, especially at the upper part of the UWB band. Therefore, detecting even small bone fractures with microwaves at a resolution of less than 1 mm is quite straightforward. Especially in the case of skull bone fracture detection, the propagation depth requirements are minimal, which enables the use of higher frequencies as well.

### 2.2. Antennas

**Figure 1:**
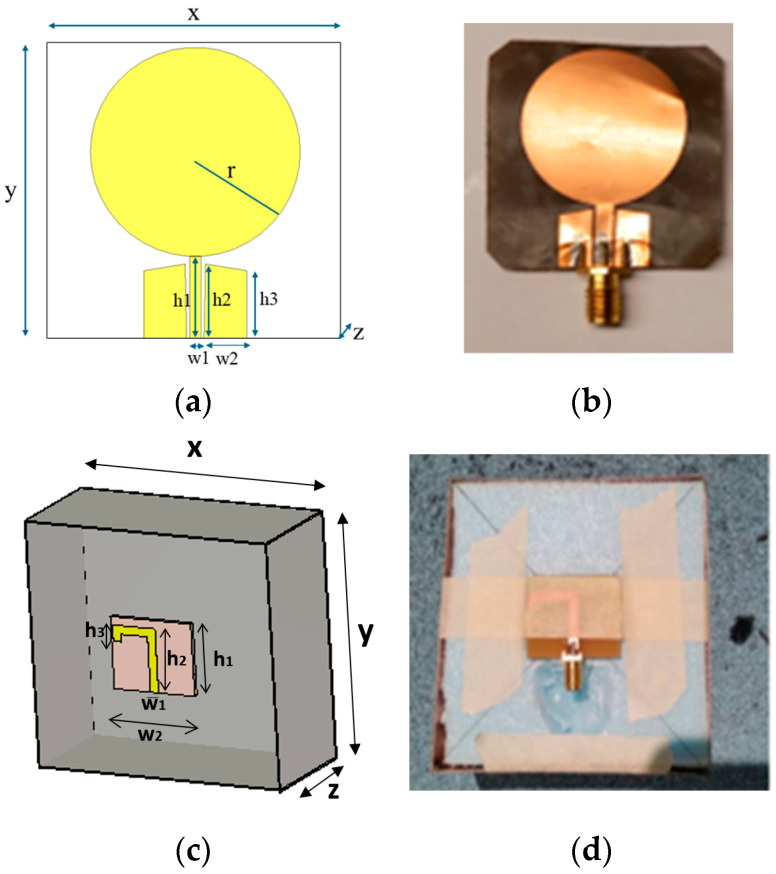
(**a**) Flexible antenna simulation model and (**b**) flexible antenna prototype for skin contact sensing. (**c**) directional cavity-backed on-body antenna simulation model. and (**d**) prototype for non-contact sensing.

The first antenna is a flexible monopole designed for in-body sensing of the 2–10 GHz frequency range, covering both the ISM band at 2.5 GHz and UWB at 3.1–10.6 GHz. The antenna is an improved version (in terms of realized gain toward the body) of the flexible antenna introduced in [26]. It is fabricated on a Rogers5880 thin flexible substrate and is designed to be attached to the skin surface. Figure 1a,b presents the simulation model and the prototype of the antenna, respectively. The dimensions of the antennas are shown in Table 2.

The second antenna is a directional cavity-backed on-body antenna designed for the lower part of the UWB band [27]. The antenna itself is small, with a size of (x, y, z = 25 × 30 × 0.75) mm, but the cavity is large, (86 × 91.5 × 39.5) mm. However, the cavity provides efficient gain towards the body. The directional cavity-backed antenna allows non-contact sensing. Figure 1c,d presents the antenna simulation model and the prototype, respectively. The radiation characteristics of the antennas are presented in Appendix A.

### 2.3. Simulation Setups

**Figure 2:**
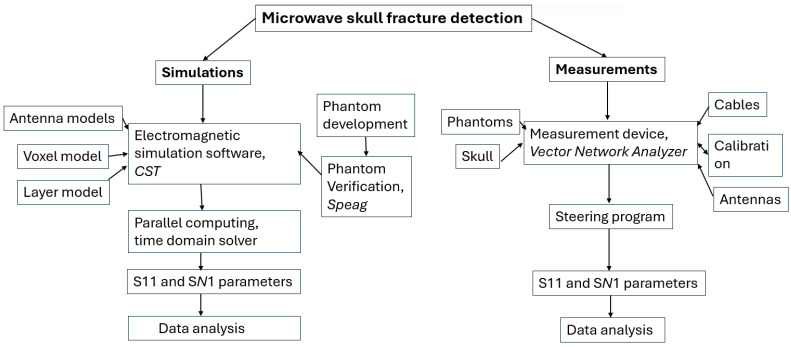
Flow chart describing microwave-based skull fracture simulation and measurement setup.

Figure 2 presents a flow chart describing the procedure to study skull fracture detection with the simulation and measurements. This subsection gives details of the simulation setup and the next subsection describes the measurement setup.

**Figure 3:**
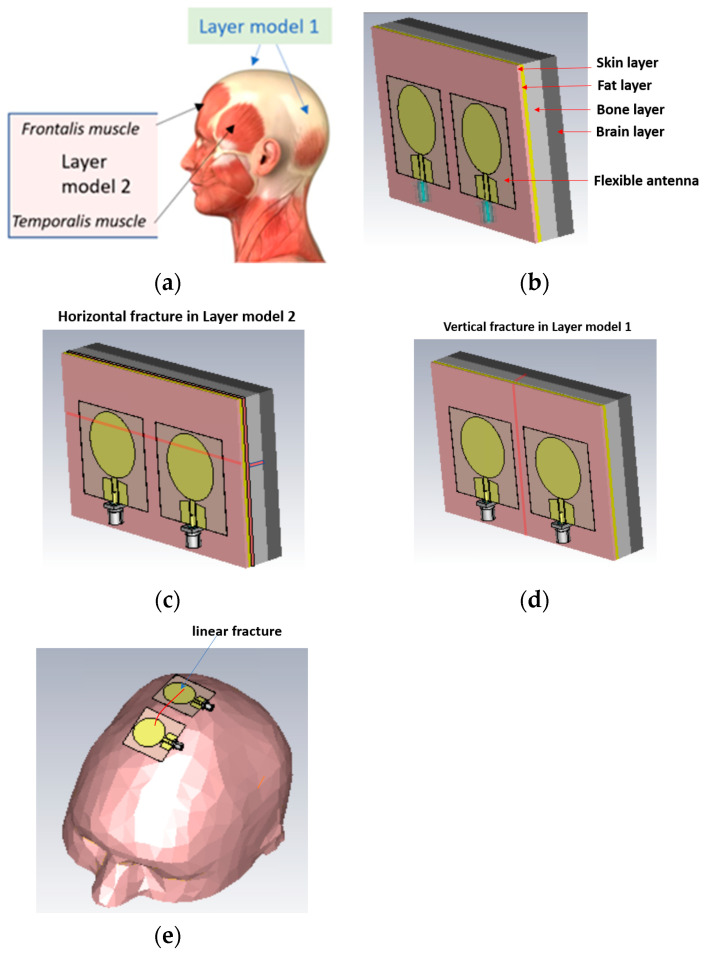
(**a**) Locations in the head for which simulation layer models 1 and 2 are valid, (**b**) layer model 1 with flexible antennas, (**c**) linear fractures in a horizontal location in layer model 2, (**d**) linear fractures in a vertical location, and (**e**) head simulation model.

The simulations were carried out with electromagnetic simulation software Dassault Simulia CST Studio Suite, version 2022 [28] using two head tissue layer models and an anatomical human head model Hugo. The dielectric properties for tissues are automatically obtained from CST’s material library, which are aligned with values presented by IT IS [20]. The first head tissue layer model (LM1) consists of skin, fat, bone, and brain layers corresponding to the locations of the head shown in Figure 3a with blue arrows. LM1 is illustrated in Figure 3b as a “reference case”, i.e., the model without fractures. The thicknesses *d* of the tissue layers are *d*_skin_ = 1.3 mm, *d*_fat_ = 1.5 mm, *d*_bone_ = 60 mm, and *d*_brain_ = 30 mm.

The second layer model, LM2, illustrated in Figure 3c is otherwise the same as LM1 but it also includes the muscle layer, hence resembling the locations including temporalis muscle or frontalis muscle, as shown with black arrows in Figure 3a. The muscle layer is between the fat and bone layer, and its thickness is 1.6 mm, i.e., the same as the average temporalis muscle layer. The average thickness of the frontalis muscle layer is 1.5 mm [1], and thus layer model 2 can be considered valid for both scenarios.

In this study, we consider simplified models for linear fracture widths of 1 mm, 2 mm, 3 mm, 4 mm, and 5 mm with horizontal and vertical locations, as shown in Figure 3c,d, respectively. In the case of horizontal fractures, both “long” and “short” fracture scenarios are evaluated. Long fractures refer to the scenario illustrated in Figure 1c, in which the fracture is located below both antennas along the whole length of the layer model, i.e., 11 cm. Short fractures refer to the scenario in which the fracture is below only one antenna, having a length of 4 cm. The length of the vertical fracture is the same as the height of the layer model, i.e., 7 cm, and it is located in the middle of both antennas. The fracture extends through the whole skull bone.

The distance between the antennas is 1 cm in most cases. Additionally, the 2 cm antenna distance is tested for comparison. The fracture is modeled as blood material, which resembles fresh skull fractures, as, e.g., in [11]. As the bone starts to heal, the fracture is modeled as connective tissue [24]. Figure 3e presents the anatomical human head model used in the evaluations. For the head model, the fracture is modeled on the top of the head. Finally, it is noted that the reference and fracture cases have the same tissue thickness, and hence the results are fully comparable.

The simulations are carried out in a time domain solver that is commonly used for voxel model simulations. Only the upper part of Hugo voxel’s head is used to save the simulation time. A frequency range of 2–10 GHz is used in the simulations with flexible antennas to enable studies on the detectability of skull fractures at different frequency ranges. With the directional antenna, the frequency range of 3–5 GHz is used since the antenna does not operate reliably in other ranges. The simulation time is accelerated using Message Passing Interference (MPI) calculation with four nodes. The total simulation time for voxel model simulations was 2 h and for layer model simulations was 20 min. As an output of the simulations, the antenna reflection coefficient S11 and channel parameter S21 are obtained.

### 2.4. Measurement Setup

**Figure 4:**
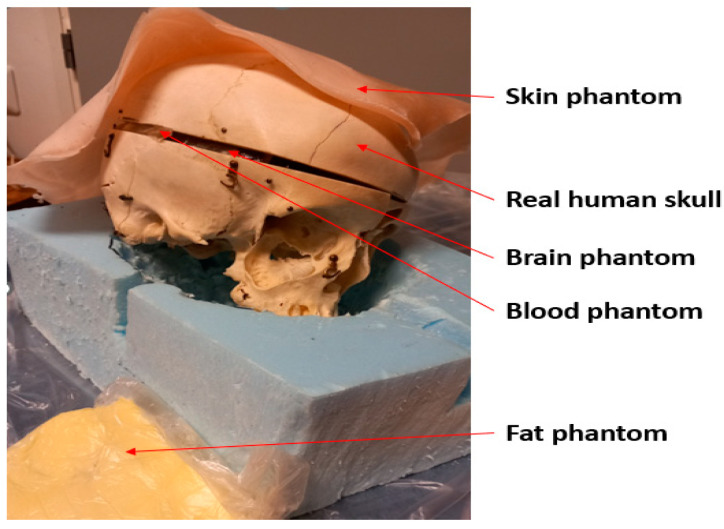
Measurement setup with a real human skull and tissue-mimicking phantoms for skin, fat, blood, and brain.

The measurements are performed using a real human skull and human tissue-mimicking phantoms prepared for skin, fat, brain, and blood tissues. The phantoms are prepared using the recipes presented in [29] using distilled water, gelatin, sugar, salt, propylene glycol, and xanthan. Phantom materials were procured from a local supermarket, except propylene glycol which was sourced from Laboratoriumdiscounter, Denmark. The dielectric properties of the phantoms were verified with a SPEAG’s probe (Zurich, Switzerland) before the measurements, which showed a good match with the average dielectric properties of human tissues [27]. Figure 4 presents the measurement setup with the skull and phantoms. Vector Network Analyzer (VNA 8720ES) of Keysight, Bochen, Germany, was used to measure antenna reflection coefficient S11 and radio channel parameter S21 for the frequency range of 2–10 GHz. The number of points in the frequency range was 1001. The VNA was calibrated accordingly before the measurements.

### 2.5. Results Analysis

In this paper, the detectability of the skull fractures is mainly analyzed by comparing antenna reflection coefficients S11/S22 or channel transfer parameter S21 between the on-body antennas in the cases of fractures having widths of 1–5 mm and in the reference case (no fractures). Additionally, power flow simulation results are presented to illustrate the impact of the facture on tissues. Power flow is defined as the time-averaged magnitude of the Poynting vector [25]. The Poynting vector quantifies the directional energy flux, representing the energy transfer per unit area per unit time within an electromagnetic field. The flux of the Poynting vector through a specified surface denotes the total electromagnetic power traversing that surface. In this context, the power flow values, expressed in decibels, have been normalized such that 0 dB corresponds to the maximum value, i.e., the value at the transmitting antenna.

Additionally, the obtained S parameters can be further used to analyze skull fractures in different signal domains, such as Black Hat transform [30], wavelet transform [31], Kolmogorov–Smirnov distance [32], etc., as well as using different methods, such as visibility graph, which converts the time series data into a graphical representation that is easier to analyze [33], Nural classifiers [34], and gradient analysis [35].

The mutual coupling between the two antennas is reduced by subtracting the scattered fields of the case when the target is absent from the scattered fields when the target is present. This is achieved by utilizing a differential Multistatic Data Matrix (MDM) approach [36].

The detection of skull fractures with different on-body antenna distances is achieved by applying singular value decomposition (SVD) on the MDM matrix. The dominant singular values are chosen for different antenna distances, which provide vital information about the properties of the skull fracture. Mathematically, the symmetric multi-static data matrix for a pair of transceiver antennas is represented as follows [37,38]:

(1) $$M=\left[\begin{array}{cc} M_{11}\left(\omega_{f}\right) & M_{12}\left(\omega_{f}\right)\\ M_{21}\left(\omega_{f}\right) & M_{22}\left(\omega_{f}\right) \end{array}\right],$$

where $M_{rt}\left(\omega_{f}\right)$ is the signal transmitted by $t^{th}$ antenna and received at $r^{th}$ antenna, $\omega_{f}\{f=0,1,\ldots ,f_{s}\}$ is the frequency of the transmitted signal, and $f_{s}$ is the total number of sampled frequencies.

The differential MDM matrix is calculated from MDM as follows [37]:

(2) $$M_{d}=M_{t}-M_{n},$$

where $M_{t}$ is the MDM matrix when the target is present and $M_{n}$ corresponds to the MDM matrix for no target (clutter).

Thereafter, applying SVD on $M_{d}$ results in [37]

(3) $$M_{d}=U\sum V^{*},$$

where U and $V^{*}$ are the unitary matrix, such as ${VV}^{*}=UU^{*}=I$; ∑ contains the singular values as diagonal elements; and ${\left(.\right)}^{*}$ is the conjugate operator. Finally, the largest singular value is chosen, which represents the response of a specific antenna configuration in detecting a particular skull fracture scenario.

To obtain more insights into the effects of the antenna configuration and position on the antenna size, another method based on the differential matrix of Root Mean Square (RMS)-based MDM is devised. The resultant matrix is termed as RMS-MDM matrix which is represented as follows:

(4) $$M_{d_{RMS}}=\sqrt{{\left(M_{t}\right)}^{2}-{\left(M_{n}\right)}^{2}}$$

Furthermore, the RMS-MDM matrix generated in Equation (4) is further utilized to compute the singular values using Equation (3).

## 3. Simulation Results

In this section, MW sensing for skull fracture detection is studied with comprehensive simulation-based evaluations carried out with layer and voxel models. Firstly, the power flow presentation is illustrated in the presence fractures to understand the fractures’ impact on propagation. Additionally, the impacts of linear fractures on the S11 and S21 parameters are studied in horizontal locations with respect to the antennas using layer models 1 and 2. The detection of the fractures is evaluated in the presence of shorter and longer fractures, as well as with two different antennas distances. Next, the healing process of the fractures is evaluated by modelling fractures with connective tissue. Moreover, the impact of linear fractures is evaluated with a vertical location. Finally, the impact of the linear fractures is investigated with an anatomically realistic head simulation model.

### 3.1. Power Flow Analysis

In this subsection, power flow representations are analyzed to understand how the fractures affect the propagation between the antennas. In this case, layer model 1 and a fracture width of 4 mm are considered for ease of illustration.

**Figure 5:**
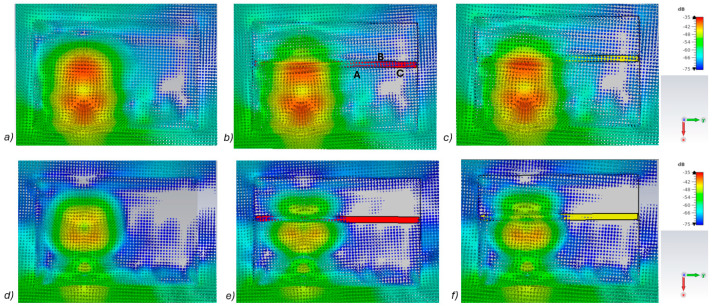
Power flow illustrations in the following cases: (**a**) reference at 5.5 GHz, (**b**) fracture filled with blood 5.5 GHz, (**c**) fracture filled with connective tissue at 5.5 GHz, (**d**) reference at 9 GHz, (**e**) fracture filled with blood at 9 GHz, and (**f**) fracture filled with connective tissue at 9 GHz.

Figure 5a–c presents power flow illustrations at 5.5 GHs for the (a) reference case (no fracture), (b) fracture filled with blood (red line), and (c) fracture filled with connective tissue (yellow line). Figure 5d–f presents the corresponding results at a frequency of 9.0 GHz. In both scenarios, the power flow illustrations are presented at the cross-section of the model corresponding to the depth of the fracture. In these figures, the dB range of −75 to −35 dB is shown, as the most significant changes were observed within this range.

As can be seen, skull fractures cause noticeable changes in power flow patterns. The additional diffractions in the fracture border are obvious due to differences in dielectric properties. Some variations are observable in the vicinity of the antenna being supplied with power but the clearest changes are visible in the area where the second on-body antenna is located. The points at which the instantaneous values of the Poynting vector are evaluated and compared across the three different cases are shown in Figure 5b, with the corresponding values presented in Table 2.

When comparing the values for reference and fracture cases, it is found that there are clear differences in Poynting vector values from 0.1 dB–6.4 dB. The changes are largest at location C at 9 GHz. Additionally, at location B, the changes are notable. These illustrations and values demonstrate the distinct advantage in utilizing two antennas for the detection of bone fractures. Interestingly, at certain locations, the power flow value is higher in the presence of fractures (e.g., location A at 9.8 GHz), whereas at other locations, the opposite is observed (e.g., location B at 9 GHz). When comparing the values for blood-filled fractures and connective tissue-filled fractures, the difference is approximately 2 dB in most evaluated cases. Generally, the power flow value for fractures filled with connective tissue is closer to the reference case, which is expected due to the smaller difference between the dielectric properties of bone and connective tissue compared to bone and blood. However, at 9 and 9.8 GHz, the power flow values for blood-filled fractures are closer to the reference values, particularly at locations B and C. The underlying cause of this phenomenon lies in the propagation characteristics at higher UWB band frequencies. Power loss in blood tissue is high at 9 GHz (higher than in connective tissue). This also results at higher frequencies in increased reflections at the blood–fracture boundary compared to the connective tissue–fracture boundary. These additional reflections constructively interfere at the receiver, manifesting as a stronger channel in the case of a blood-filled fracture compared to a connective tissue-filled fracture.

It should be noted that this power flow is a 3D illustration and Figure 5 is only an example case of one cross-section at the fracture depth. The power flow values differ within cross-section and locations. However, the presented power flow illustration and value comparison gives an insight into how a fracture causes additional scattering and diffractions, which affect the overall power flow either positively or negatively compared to the reference case.

### 3.2. Linear Fracture in the Horizontal Location

Firstly, the impacts of the long, horizontally located skull fractures on the S11 and S21 parameters are studied using layer models and flexible antennas. The simulations are carried out with skull fracture widths of 1 mm–5 mm and with the reference case. In addition, the effect of the distance between the antennas is studied by conducting simulations with the antennas spaced 1 cm and 2 cm apart.

#### 3.2.1. Case 1a: Layer Model 1 with a Long Horizontal Fracture

**Figure 6:**
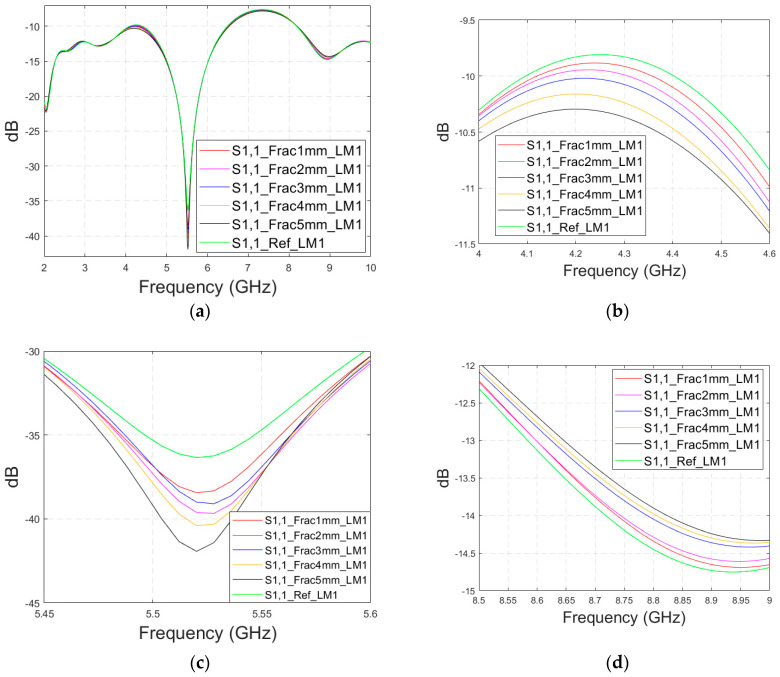

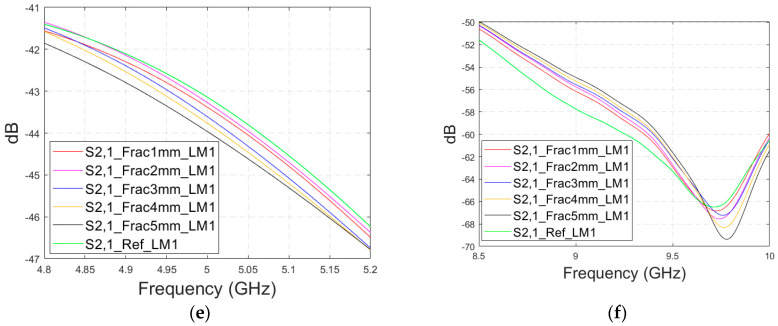
Simulated S11 and S21 results for Case 1 (layer model 1 with long horizontal fractures and flexible antennas with a 1 cm distance): (**a**) S11 results for fracture widths of 1–5 mm for the whole simulated frequency range and (**b**) zoomed to 5.4–5.6 GHz, in which the differences are the largest, (**c**) S11 at 4–4.6 GHz, (**d**) S11 at 8.5–9 GHz, (**e**) S21 at 4.8–5.2 GHz, and (**f**) S21 at 8.5–10 GHz.

Firstly, the impacts of the long, horizontally located skull fractures on the S11 and S21 parameters are studied using LM1 and flexible antennas with a distance of 1 cm. The simulations are carried out with skull fracture widths of 1 mm–5 mm and with the reference case. The S11 parameters with a 1 cm antenna distance are shown in Figure 6a for the whole simulated frequency range *f* = 2–10 GHz and in Figure 6b for the zoomed version for the value of 5.5 GHz at which the differences are at largest in this case. Additionally, S11s for the ranges of 4–4.6 GHz and 8.5–9 GHz are shown in Figure 6c,d, respectively.

It is found that the differences in S11s are relatively minor except at 5.3–5.5 GHz, in which the S11’s notch depth increases systematically with the growth of the fracture width. The difference between the S11 of the reference case and the S11 of the 1 mm fracture case is 2 dB, whereas for the 5 mm fracture case, the difference is even 6 dB. These kinds of changes are easily detectable in practical measurements. The impact of the fracture is similar at 4–4.6 GHz. The S11 level decreases as the fracture width increases. Instead, at higher frequencies of 8.5–9 GHz it is vice versa; the S11 level increases as a function of the fracture width. The reason for the different tendencies can be found in the differences in dielectric properties between the bone and blood tissues, as shown in Table 1. At lower frequencies, the differences in relative permittivity are more significant, whereas at higher frequencies the changes in conductivity values increase clearly.

Next, channel parameter S21 is evaluated for the same scenario. The results are presented in Figure 6e for the whole frequency range. The reference case is clearly at a higher level at 2–3.5 GHz; the difference is up to 5 dB. However, the difference between the different fracture widths is minor. It is found that the fractures are best detectable at around 5 GHz and 9.8 GHz, for which the zoomed versions are presented in Figure 6e,f, respectively. The level of the S21 parameter is higher in the reference case than in the fracture case around 5 GHz. The difference between the widest fracture of 5 mm and the reference case is 3 dB. A fracture width of 1 mm causes a 1 dB difference in the S21 parameter, which is still detectable. Instead, at 9.8 GHz, S21 for the reference case is at the highest level: the difference between the fracture cases is 2–4 dB. Along with the power flow evaluations, these results prove that the detectability of skull fractures is frequency dependent and the impact of the fractures.

#### 3.2.2. Case 1b: Layer Model 2 with a Long Fracture

In this subsection, we investigate the detection of horizontal fractures with layer model 2 (LM2), which corresponds to the areas in the head with the muscle layer (as shown in Figure 3c). Likewise in Case 1a, flexible antennas are used in the evaluations. The muscle layer has high relative permittivity, as shown in Table 1, and hence, a higher propagation loss. Therefore, the muscle layer is expected to have a detrimental effect on the detection accuracy.

**Figure 7:**
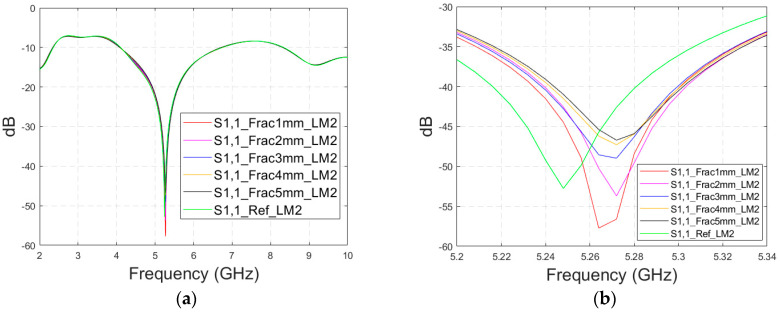

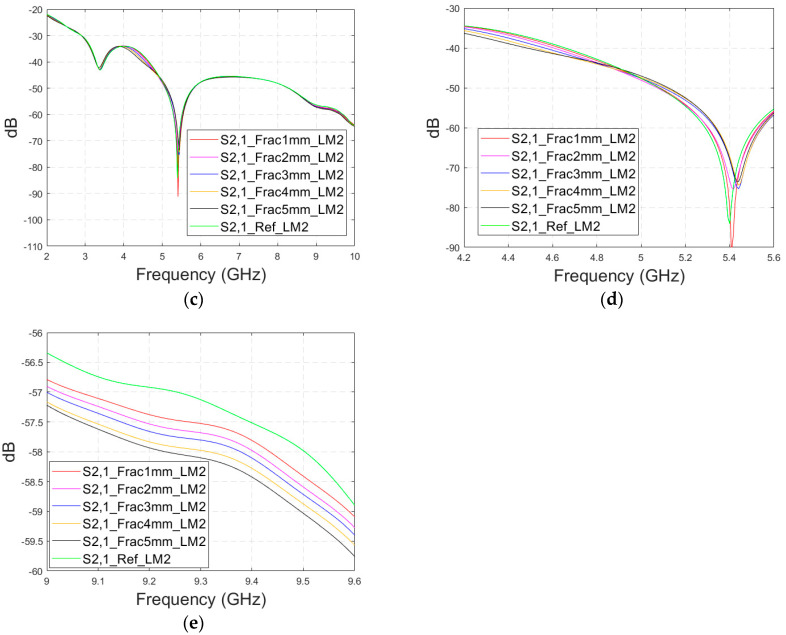
Simulated S11 results for Case 1b (layer model 2 with horizontal fractures): (**a**) S11 results for the whole frequency range, (**b**) S11 results zoomed for 5.25–5.34 GHz, (**c**) S21 results for the whole range, (**d**) S21 results for 4.2–5.6 GHz, and (**e**) S21 for the 9–9.6 GHz range.

The simulated S11 parameters are shown for the whole frequency range in Figure 7a and zoomed version in Figure 7b for the range of 5.2–5.34 GHz, in which the differences are the most detectable. It is also shown that in the presence of muscle layer, the smallest fractures are visible, although the difference between the smallest fractures is minor than with layer model 1. The differences in the S11 parameter are not detectable above 6 GHz due to a decreased penetration depth at higher frequencies. Instead, the differences in the S21 parameter, which are presented in Figure 7c–e, are detectable also at 9–10 GHz, as in the case of layer model 1. However, the differences due to the fractures are minor.

**Figure 8:**
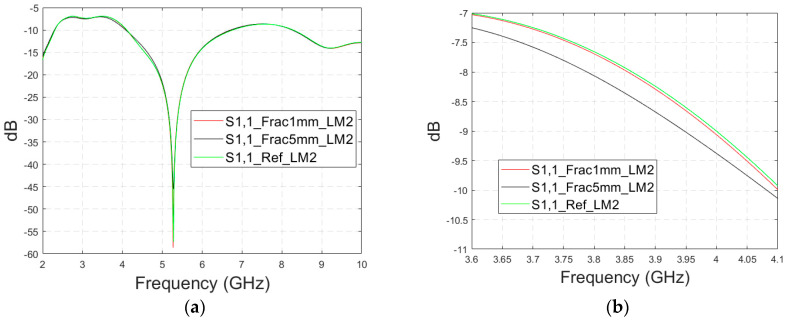

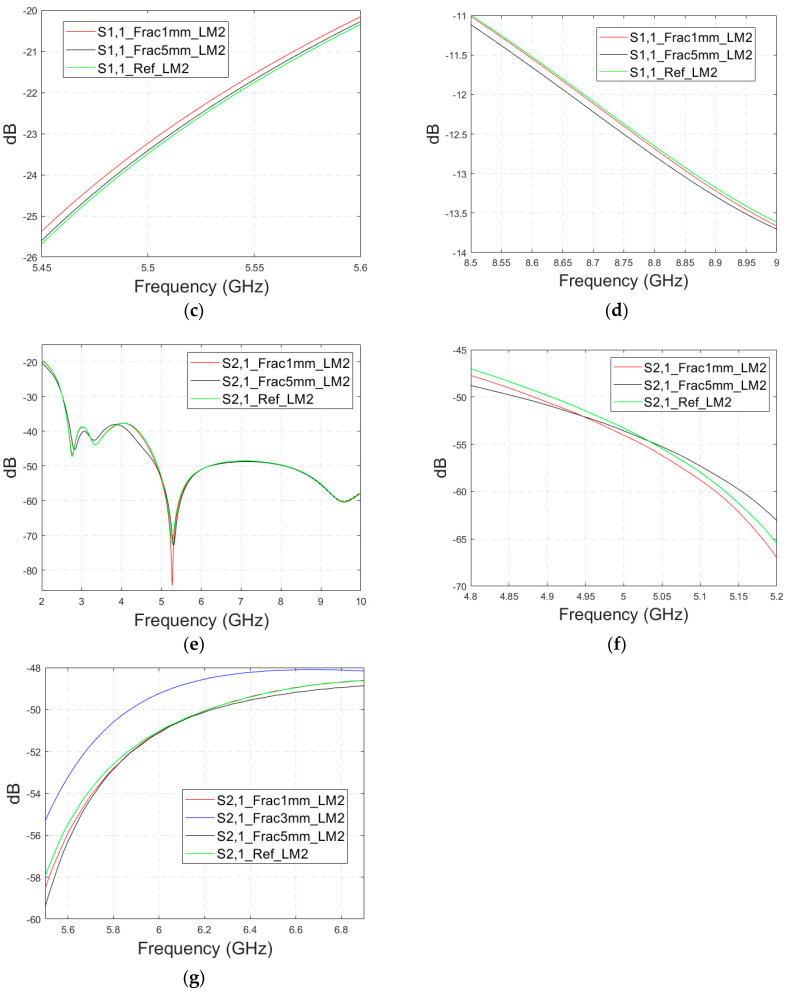
Simulated S11 and S21 results obtained using layer model 2 with horizontal fractures and a 2 cm antenna distance (**a**) S11 results for the whole frequency range, (**b**) S11 results zoomed for 3.55–4.15 GHz, (**c**) S11 results zoomed for 5.45–5.6 GHz, (**d**) S11 results zoomed for 8.5–9.0 GHz, (**e**) S21 results for the whole range, (**f**) S21 results for 4.8–5.2 GHz, and (**g**) S21 for the 5.55–6.85 GHz range.

Next, the impact of the distance between the antennas is evaluated using LM2 and a distance between the flexible antennas of 2 cm (Case 1c). The S11 and S21 results are shown in Figure 8a,b. It can be seen that the detectability of skull fractures clearly improves as the antenna distance increases. This is due to the decrease in the antennas’ mutual coupling as the distance increases.

**Figure 9:**
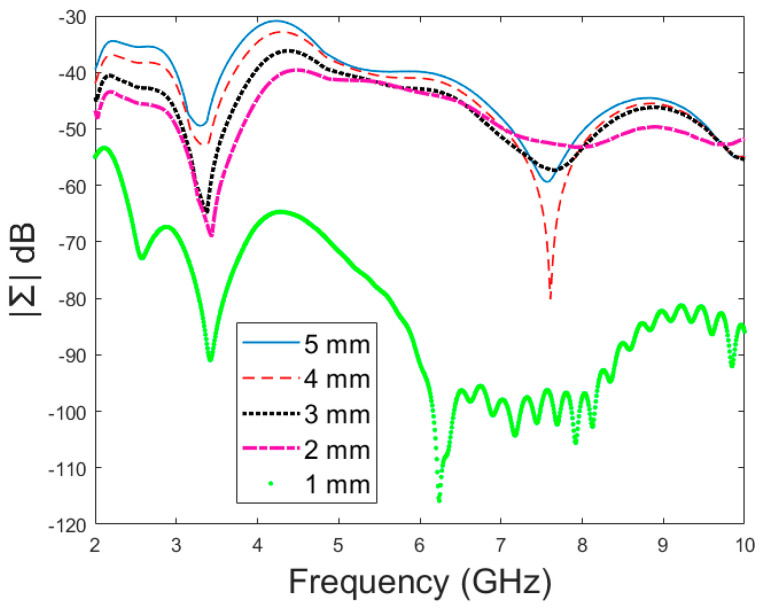
Amplitude (dB) vs. frequency (GHz) of the dominant singular value of MDM for different fracture sizes.

**Figure 10:**
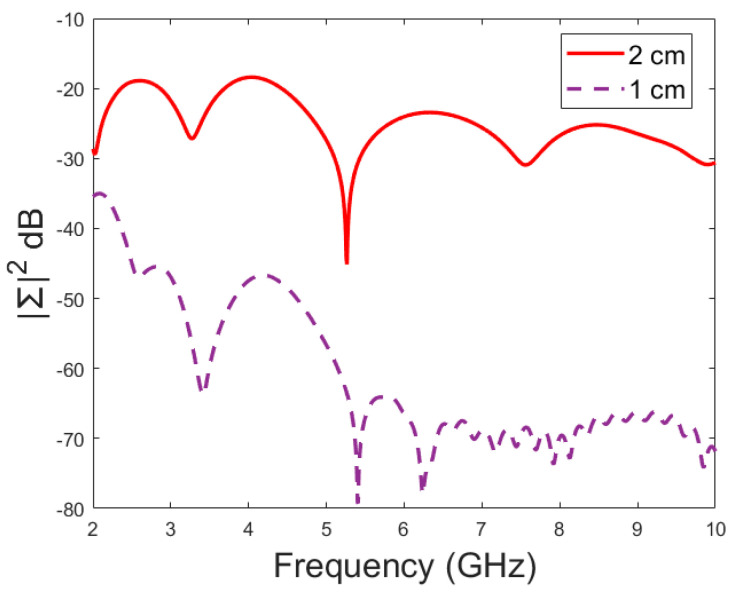
Amplitude (dB) vs. frequency (GHz) of the dominant singular value of RMS-MDM for different antenna configurations.

Next, the detectability of skull fractures is compared using the MDM matrix and RMS-MDM matrix approaches described in Section 2.5. It can be visualized from the MDM and RMS-MDM matrix-based results that the detectability of skull fractures increases as the antenna distance increases. The difference between the different fracture conditions and antenna configuration is clearer in these results compared to the conventional S11 and S21 results. It can be visualized in Figure 9 that the amplitude of the dominant singular value of MDM (Σ) for a 5 mm fracture size is the largest and that with a 1 mm fracture size is the smallest. The effect of the antenna distance is visualized in Figure 10, wherein the amplitude of the dominant singular values of the RMS-MDM matrix are plotted for 1 cm and 2 cm antenna configurations. Although a larger distance between the antennas yields better detectability due to smaller antenna coupling, the benefit of using a smaller antenna distance is justified to enable the detection of shorter fractures also from the S21 parameter. The smaller the distance between the antennas, the more likely it is that shorter fractures will occur below both antennas when searching for a fracture location. The evaluation results for LM1 and short fractures are presented in Appendix A as Case 1c.

#### 3.2.3. Case 2: Layer Model 1, Fracture Healing Process Evaluations with Connective Tissue

As discussed earlier, the fresh fractures are conventionally modelled with a blood-filled gap in the bone tissue. However, as time passes and the healing process starts, the fracture can be modelled with connective tissue.

**Figure 11:**
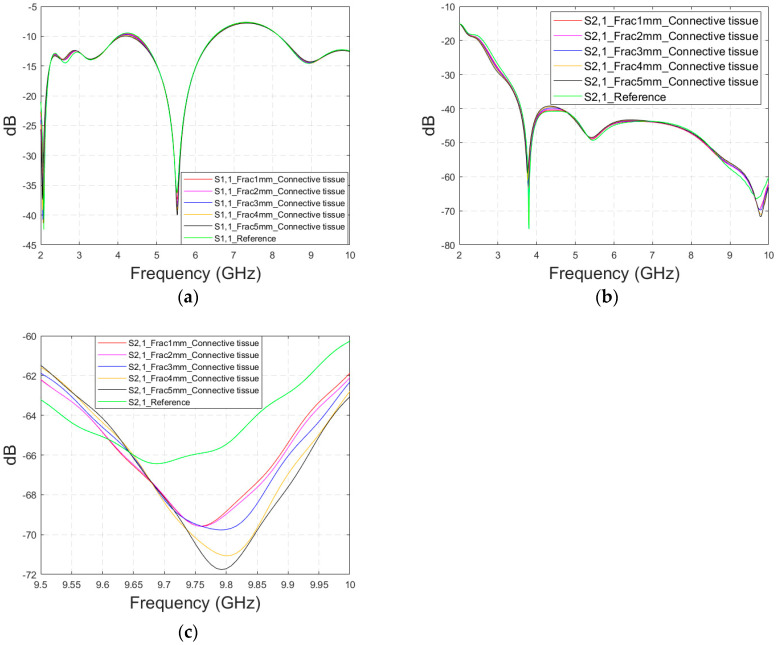
Simulated S11 and S21 results obtained using flexible antennas and layer model 1 with horizontal fractures filled with connective tissue: (**a**) S11 results with fracture widths of 1–5 mm for the whole simulated frequency range, (**b**) S21 results for the whole frequency range, and (**c**) S21 zoomed at 8.5–10 GHz.

In this subsection, we present S11 and S21 evaluations for LM1 in which the fracture is filled with connective tissue instead of blood. The results for the S11 parameter are shown in Figure 11a and those for the S21 parameter are shown in Figure 11b,c. The tendency is found to be similar as the fracture gap is filled with blood, as shown in Figure 6a–f for Case 1a. However, the differences between the reference and fracture cases are minor in the case of S11 results obtained with connective tissue. For instance, around 5.5 GHz, the S11 difference between the reference and 5 mm fracture cases is 4 dB with connective tissue, whereas at the same frequency range, the difference with blood was 5.8 dB. The reason for the better detection of the blood-filled fracture than connective tissue-filled fracture is the larger difference in dielectric properties between the bone and blood than between bone and connective tissue, as shown in Table 1. However, interestingly at 9.8 GHz, it is vice versa; differences between the reference and fracture cases are slightly larger with the connective tissue than with the blood. The channel attenuation is approximately 2 dB higher in the case of connective tissue than with the blood for all the studied fracture widths. At higher frequencies, the differences between the dielectric properties are smaller, as seen in Table 1. Thus, the reason for this phenomenon can be found in propagation characteristics at higher frequencies: the relative permittivity of blood is higher than connective tissue and thus this causes more reflections in the blood–fracture border at higher frequencies than in the connective tissue–fracture border as the signal travels from antenna 1. These additional reflections are summed positively in the receiver, which can be seen as a stronger channel than in the case of connective tissue fracture. This result is also aligned with the power flow values presented in Table 3.

#### 3.2.4. Case 3: Realistic Head Simulation Model with a Longer Fracture

**Figure 12:**
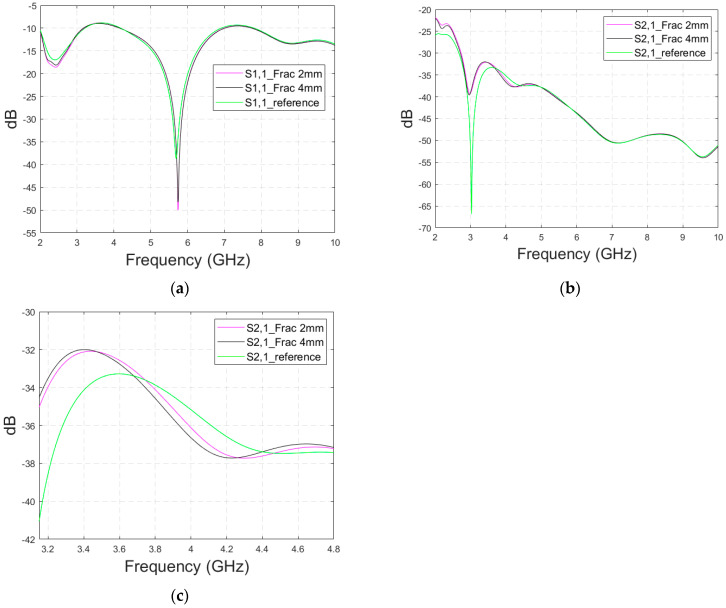
Simulated S11 results obtained using the anatomical head simulation model with long horizontal fractures filled with blood: (**a**) S11 results for the whole frequency band, (**b**) S21 results for the whole frequency band, and (**c**) S21 results for 3.15–4.8 GHz.

In this subsection, the impact of long fractures is evaluated using a head simulation model of CST’s Hugo voxel. The head model has an anatomically realistic shape, except that the layers below the skin are modelled as an averaged muscle–fat layer. Additionally, its thickness is almost 30% larger than in a realistic scenario. The fracture is located similarly with respect to the antennas, as in the case of layer models. In this case, only fracture widths of 2 mm and 4 mm are evaluated to save simulation time. The S11 results are shown in Figure 12a and the S21 results are shown in Figure 12b,c. Moreover, in this case, the effect of the fracture can be seen as either an increased or decreased antenna reflection coefficient or channel parameter, depending on the frequency. For instance, the S11 value is at a lower level in the reference than in the fracture case at 4.5–5.7 GHz, and otherwise the fracture cases are at the lower level.

In general, the differences are smaller than in the case of the layer model since the thickness of the tissues above bone layer is significantly larger. However, at the notch area of 5.8 GHz, the difference between the fracture and reference case is relatively large at −12 dB. In this case, a 2 mm fracture width yields a difference larger than 4 mm, which is contradictory. Nevertheless, although the fracture width cannot be distinguished from the data, it shows a clear difference from the reference case.

With the S21 results, the difference between the reference and fracture cases is visible until 5.8 GHz, with the clearest difference of 27 dB at 3 GHz. The difference is large since S21 has a deep notch at 3 GHz, which is smaller with fracture cases. Figure 12c illustrates clearly how the impact of the fracture may change with frequency; from 3–3.7 GHz, the S21 level is lower in the reference case than in the fracture case, whereas from 3.7–4.4 GHz it is vice versa. Similarly, the differences fluctuate until 5.8 GHz, after which no differences are visible.

Appendix A (at the end of this paper) presents evaluations with the realistic head model and shorter fractures (Case 3b). Additionally, the appendix presents results for a modified head model in which the average muscle–fat tissue layer under the scalp is replaced with a pure fat layer. This model, Case 3c, corresponds more to the realistic scenarios in the locations without muscle layers, e.g., on the top of the head.

### 3.3. Linear Fracture in a Vertical Location

#### Case 4: Layer Model 1, Long Fracture in a Vertical Location

**Figure 13:**
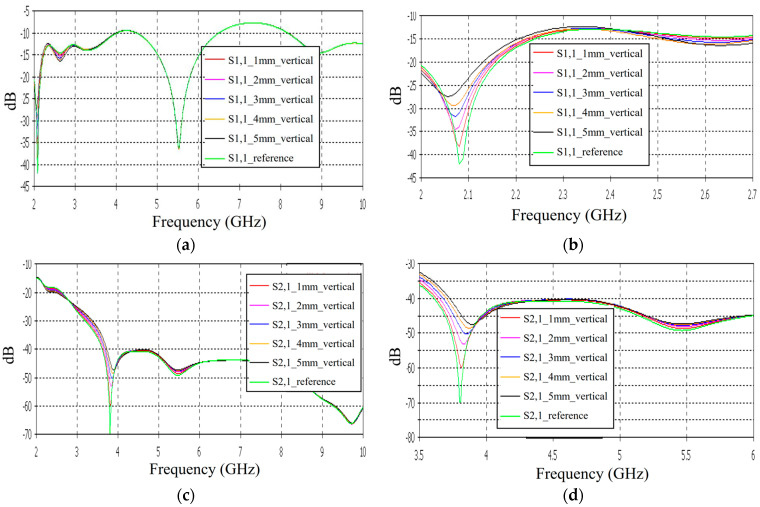
Simulated S11 and S21 results obtained using flexible antennas and layer model 1 with vertical fractures filled with blood: (**a**) S11 results obtained with fracture widths of 1–5 mm for the whole simulated frequency range and (**b**) zoomed to 2–2.7 GHz in which the differences are the largest, (**c**) S21 for the whole frequency range, and (**d**) S21 at 3.5–6 GHz.

In this subsection, we evaluate the impact of a skull bone fracture in a vertical location with respect to the antennas, as shown in Figure 1d Layer model 1 and the flexible antennas are used in the simulations. The S11 results obtained in the presence of vertical fractures having widths of 1–5 mm are presented in Figure 13a for the whole frequency range and in Figure 13b for the zoomed version for 2.45–3.5 GHz. Additionally, in this case, clear changes can be seen in the S11 parameter, although the antenna is not located directly above the fracture. Around 2.08 GHz, in which the changes are most visible, the level of the S11 parameter increases as the fracture increases. The difference between the reference and 1 mm fracture case is even 4 dB and the reference and 5 mm fracture case is 16 dB. Instead, at 2.5–2.7 GHz, the order is vice versa: the S11 parameter decreases as the fracture increases. In this case, the differences are smaller, with a maximum of 2 dB.

The channel parameter S21 is shown in Figure 13c for the whole frequency range. In this case, the clearest differences can be seen at 2.45–4 GHz and 5.4 GHz. In this case, the largest differences can be seen at 3.8 GHz, in which the 1 mm fracture causes a 10 dB difference and the 5 mm fracture causes a 24 dB difference in the S21 results. The results are promising, as even small fractures can be detected with both S11 and S21 measurements when positioned vertically between the antennas, without being directly beneath either one.

### 3.4. Cavity-Backed Antenna Evaluations

The previous subsections presented evaluations with flexible antennas that are designed to be on the skin surface. Next, the impact of skull fractures is evaluated with a directional cavity-backed antenna (CA) that can be used both in contact and non-contact sensing. Evaluations are carried out using LM1 with blood-filled horizontal fractures having widths of 1 mm, 3 mm, and 5 mm. In this case, only the frequency range of 3–5 GHz is simulated due to the antenna’s limited operational bandwidth.

#### 3.4.1. Case 5 Layer Model 1, Cavity-Backed Antenna with a Distance of 23 mm

**Figure 14:**
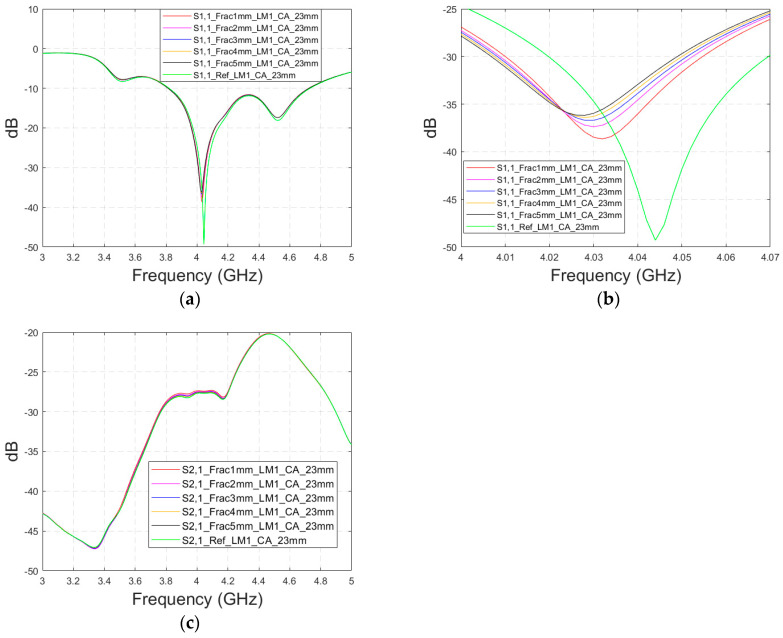
Simulated S11 and S21 results obtained using layer model 1 and cavity-backed antennas with a 23 mm antenna–skin distance: (**a**) S11 results obtained with fracture widths of 1–5 mm for the whole simulated frequency range and (**b**) zoomed to 4–4.07 GHz in which the differences are the largest, and (**c**) S21 results for the whole frequency range.

This subsection presents skull fracture evaluations with non-contact sensing using a cavity-backed antenna with a 23 mm distance, which is more optimal for the operationality of this antenna. These evaluations are carried out with layer model 1. The S11 results are shown in Figure 14a,b for the whole frequency bandwidth of 3–5 GHz and zoomed version at 4–4.07 GHz, respectively. As can be seen, the fractures cause larger differences in the S11 results: the difference between the reference case and the 1 mm fracture case is 12 dB and that of the 5 mm fracture case is 14 dB.

In the S21 results, which are presented in Figure 14c, small changes can be seen in fracture cases in the frequency range of 3–4.4 GHz. However, the changes are so small, approximately 0.5 dB, which are more challenging to detect. Thus, only the S11 results are evaluated in the next cases with cavity-backed antennas.

#### 3.4.2. Cases 6–7 Head Models with a Cavity-Backed Antenna and 0 mm and 23 mm Antenna–Skin Distances

**Figure 15:**
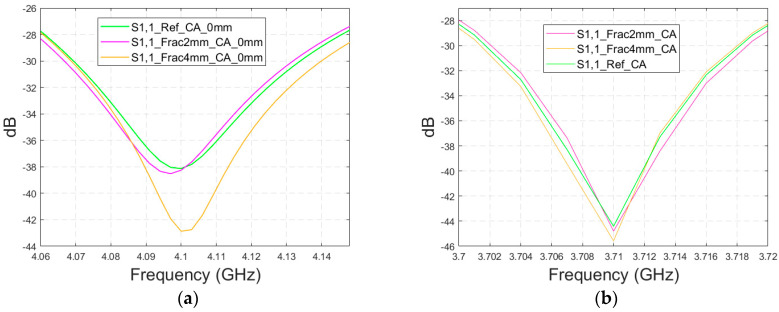
Simulated S11 obtained using the realistic Hugo head model and cavity-backed antennas with (**a**) a 0 mm antenna–skin distance and (**b**) 23 mm antenna–skin distance.

Finally, the impact on the skull fractures is evaluated using cavity-backed antennas and CST’s Hugo voxel head model. Firstly, the antenna–skin distance of 0 mm is evaluated. In this case, only fracture widths of 2 mm and 4 mm are investigated to save simulation time with the more complex voxel model. The values of the S11 parameter for the reference and fracture cases are presented in Figure 15a. In this case, the largest difference, 5 dB, between the reference and the 4 mm fracture case can be seen at 4.1 GHz. The maximum difference between the reference and 2 mm fracture case is 1 dB at 4.09 Hz. Due to the large size of the cavity-backed antenna, the S21 results are not evaluated with the realistic model. The placement of the second antenna would need some evaluations and verification, which have been left for future work.

With an antenna–skin distance of 23 mm, as presented in Figure 15b, the difference between the reference and fracture cases is largest, even 7.51 dB, at 3.94 GHz. The difference between the 2 mm and 4 mm fractures is negligible in this case, which is the opposite of Case 5, where the difference between the reference and 2 mm fractures was minor, but the difference between the reference and 4 mm fractures was significant.

## 4. Experimental Results

In this section, the impact of the skull fracture is evaluated using a human tissue phantom prepared with skin, fat, brain, and blood phantoms [29], as well as a real human skull. Both flexible and cavity-backed antenna prototypes are used in the evaluations. The measurement results are compared with the simulation results obtained using layer model 1 and a horizontal fracture (the tissue thicknesses are similar), as well as with the simulation results obtained with the Hugo head model (the shape is similar).

### Cases 8–9: Human Skull and Phantom with Flexible Antennas and Cavity-Backed Antennas

**Figure 16:**
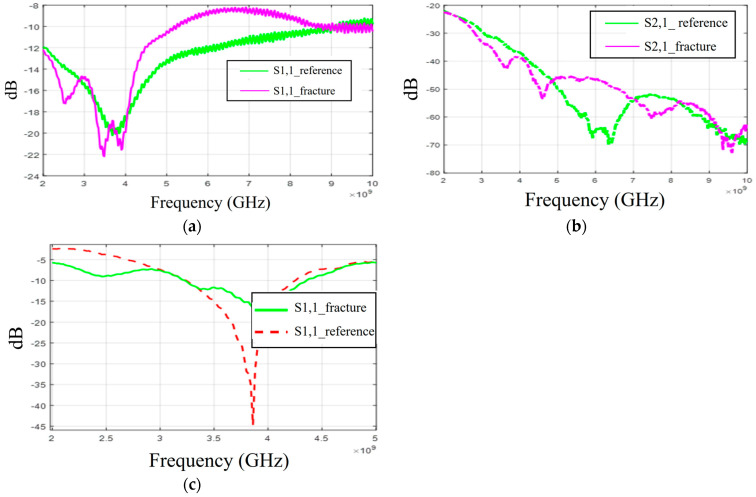
Measurement results obtained with a real human skull and tissue phantoms: (**a**,**b**) S11 and S21 results obtained with flexible antennas in the presence of a 2 mm fracture and (**c**) S11 results obtained with the cavity-backed antenna.

Firstly, the S11 results obtained with flexible antennas are presented in Figure 16a. Similar to the simulation results, the impact of the fracture varies with the frequency. In the measured case, S11 of the fracture case is at a higher level at around 3 GHz and 4–8.5 GHz. In the S21 results presented in Figure 16b, the reference case is at the lowest level at 6–7 GHz and 8–9 GHz. Instead, at 9.5 GHz, S21 of the reference case is at a higher level, as in the simulation results for Case 1a.

When comparing the measurement and simulation results, one can note a similar tendency, especially in the S11 results at frequency ranges around 5 GHz and 9.5 GHz. At around 5 GHz, the fractures have a higher level of S11 results than in the reference case, and at 9.5 GHz, they are vice versa. In the S21 results, a similar tendency can be found with the measurement and all corresponding simulation results, especially in the 2.5–3 GHz and 4 GHz ranges, where the impact of the fractures can be seen as a decreased S21 parameter.

Finally, the measurement results with a cavity-backed antenna are presented in Figure 16c. In this case, the antenna–skin distance is 0 mm and the fracture width is 2 mm. The measured S11 results, presented in Figure 16c, show that the level of the S11 parameter is lower in the fracture case than in the reference case at the antenna’s operational frequency range. This trend aligns with the observations from both the layer model and the Hugo head simulation model results. However, the discrepancies between the fracture and reference cases are more pronounced than in the simulations. Additionally, there is a minor frequency shift in the S11 notch area. These differences arise because the simulation and measurement models differ in terms of model shapes and tissue thicknesses. Furthermore, the antenna’s sensitivity to skin contact contributes to these variations. When the phantoms are attached to a real human skull, achieving smooth skin contact is more challenging, particularly with flexible antennas. In practical scenarios, where the antennas would be embedded within a measurement device (e.g., a measurement band or helmet), they would be securely attached to the skin surface.

Table 4 summarizes the simulation and measurement results for different scenarios, highlighting the frequency ranges where differences in the S11 and S21 results due to fractures are most noticeable. It also indicates the extent of the fracture’s impact on the results and whether this impact is increasing or decreasing for the S11/S21 response levels. In the various cases studied, skull fractures are most detectable around 4–5 GHz and 9 GHz. At 4–5 GHz, the S21 level increases with the fracture width, whereas at 9.5 GHz, the S21 level decreases as the fracture width increases.

From Table 1, it is evident that at 5–6 GHz, the differences in the dielectric properties of bone tissues and blood are significant in both relative permittivity and conductivity values. The relative permittivity and conductivity values of blood are approximately 450% higher than those of bone. Similarly, at 9.5 GHz, the differences are notable, particularly in conductivity, where the conductivity of blood is about 525% higher than that of bone.

## 5. Discussion

The results of the comprehensive skull fracture evaluations showed that fractures cause clear differences in antenna and channel parameters in most of the scenarios. In the S11 results, the difference is 0.1–20 dB, and in the S21 results, the difference is 0.1–30 dB. Even the smallest 1 mm fractures are visible with both the S11 and S21 parameters in most of the studied cases, especially if, e.g., an MDM approach is used to reduce coupling between the antennas. In most cases, the fracture changes S parameters logically: either the level increases or decreases with the width of the fracture, depending on the frequency. Additionally, there are also some frequencies in which the differences due to skull fractures are almost negligible. There are several reasons that may have impact on this tendency since the interactions between electromagnetic waves and human tissues are complex phenomena. As explained in Section 2, when a signal encounters the boundary between two tissues with different dielectric properties, it may undergo reflection, scattering, and transmission. These effects can combine in various ways, either enhancing or diminishing the signal received by the antenna. Additionally, the characteristics of the antenna itself influence the overall propagation pattern. Therefore, in certain frequency ranges where no differences are observed between the reference and the fractures cases, it is likely that the antenna’s radiation characteristics are not optimal in terms of detectability and the signal components caused by the fractures may be summed in such a way that they are not visibly distinguishable. Power flow illustrations also support this consideration; at certain areas and frequencies, the differences between the power flow values of reference and fracture cases are remarkable, whereas at some areas/frequencies, the differences are negligible.

The varying impacts of abnormalities in S parameters with the frequency have also been found in several other microwave technique-based studies presenting results with a larger frequency band, e.g., in stroke detection, breast cancer detection, brain tumor detection [19], and intracranial pressure monitoring [39]. However, most of the studies select only the frequency band in which the changes in the S parameters due to abnormalities are either positive or negative, which is of course the aim for the practical scenario. Power flow illustrations provide insights into why such phenomenon may occur. At certain frequencies, additional diffractions due to fractures may sum either positively or negatively in the receiving antenna side, depending on the location of the fracture with respect to the antennas, as well as the frequency. Nevertheless, more comprehensive studies are needed utilizing different antennas that operate at the same frequency bands but have varying radiation characteristics, and by altering the position of the fracture relative to the antennas.

There is a clear correspondence in skull fracture detection trends between the simulation results and experimental results. However, in these evaluations, skull fractures are more easily detected in the measurement results. One reason for this is that the real human skull has dried over time, leading to a decrease in its relative permittivity. Consequently, the difference in dielectric properties between the skull bone and the blood phantom is greater than in the simulations. Additionally, the antenna’s contact with the skin phantom in the experiments may cause differences in the results. Even small changes in the on-body antenna’s skin contact or the antenna–skin distance can significantly affect performance, as discussed in [40]. In general, the difference in simulation and measurement results are sometimes inevitable, especially in more complex setups, for several reasons. For instance, the dielectric properties of materials and in general models used in simulations may not perfectly match those used in measurements, leading to discrepancies. The setup for measurements, including the positioning of antennas and the presence of nearby objects, can introduce variables that are difficult to replicate identically in the simulations. Moreover, the characteristics of antenna prototypes are seldom identical to those of the simulation model, yielding differences in results.

In this paper, the evaluations with a flexible antenna were shown for a frequency range of 2–10 GHz to enable studies of the most optimal frequencies for skull fracture monitoring. However, it is emphasized that for realistic implementation, the use of such a wide frequency band of 2–10 GHz is neither expedient nor practical. It will be essential to select the range in which the impact of the skull fractures is most visible and the effect of skull fracture widths is logical. Based on these results, frequency ranges around 4–5 GHz and 9.5 GHz appear to be most promising for fracture monitoring. However, this initial assessment still needs to be verified with other antennas as well, which is left for future work. Additionally, it is important to understand the impacts of the antenna characteristics on the detectability of skull fractures. Thus, our future studies will also include evaluations with varying antenna parameters and different antennas having different radiation characteristics.

It is noteworthy that the simulations are carried out using dielectric properties of the average human tissue given in [21]. Similarly, the average thickness of head tissues is used. However, both dielectric properties and tissue thicknesses may vary between individuals, which can affect the methodology. One of our next studies will include evaluations in which both the dielectric properties and tissue thicknesses are varied according to the natural variation range. It is essential that actual reference data sets in practical implementations are generated using the information on natural variations between individuals both in dielectric properties and thicknesses. In practice, this will require the use of efficient machine-learning algorithms. Additionally, the S11 and S21 parameters must be measured also on the healthy area of each measured patient to facilitate the selection of a reference data set. More comprehensive considerations of how reference data sets will be formulated will also be left to future studies.

For practical implementation, the sensitivity of the measurement device plays a crucial role in detecting skull fractures. In the measurements conducted for this study, an accurate VNA with a low noise level was used and thus the skull fractures were clearly visible. In practice, a highly sensitive measurement device with a low noise level is required. Additionally, the detectability of the skull fractures could be enhanced by different channel analysis methods in different domains [11] or by embedding a predictive neural network platform [37]. These studies will also be left for future work.

In this study, the Tx and Rx antenna distance is only 1–2 cm to enable the detection of even shorter skull fractures in the S21 parameter analysis. As one of the future study cases, we will study the impact of the antenna distance on the detectability of the skull fractures more comprehensively.

The main novelty of this study is that it presents comprehensive evaluations of microwave sensing-based skull fracture detection/skull fracture healing process monitoring with realistic models corresponding to different areas in the head. This is also the first paper presenting assessments on frequency selection for microwave-based skull fracture monitoring, showing also the power flow-based analysis.

The limitations of this study, encompassing both simulations and measurements, are partially addressed in the discussion of future work but are briefly summarized here. (a) The study utilizes phantoms with dielectric properties representative of average human tissue in both simulations and measurements. (b) Advanced data analysis methods are not used within this paper and are deferred to future research. (c) The investigation is confined to two different antennas; incorporating a broader range of antennas would yield more comprehensive insights into the phenomenon, and (d) the use of a dry human skull, as opposed to a realistic skull phantom, for microwave studies. Our next study will address these limitations.

The possibility of detecting skull fractures with a portable device, e.g., in the ambulance, would bring several advantages and speed up the start of the treatment. Additionally, information on possible skull fractures is essential for MW-based stroke detectors, which can also be based on measuring channel parameters between the antennas on the head. This is due to the fact that both skull fractures and strokes have impacts on the channel characteristics and partially in the same frequencies [19]. Thus, the possible skull fractures should be first detected and analyzed so that they can be taken into account in a stroke detection analysis to avoid false alarms. In principle, it would be beneficial if the same devices could be used for both skull fracture detection and stroke detection. Especially in the case of injuries involving large blows to the head, which may cause both skull fractures and hemorrhagic strokes, it is relevant to know which of these injuries are involved.

Besides skull fracture detection, MW sensing-based devices may enable a safe and simple method to evaluate the healing progress of skull fractures. Our evaluations show that the fractures filled with connective tissue, which resemble the fractures in the healing process, are also easily detectable at different fracture widths. Due to safety, the MW technique can be used for frequent measurements and hence enables continuous monitoring of the healing process.

Our future work includes studies for MW-based bone fracture detection in different locations using several antennas operating at distinct frequencies. Furthermore, we will study different channel parameter analysis methods in different domains to maximize the detectability of bone fractions, which is especially important when monitoring the healing process of the fractures. Moreover, clinical studies with skull fracture patients will be included in our next steps. We also plan to continue our stroke detection studies by evaluating strokes in the presence and absence of different skull fractures.

## 6. Summary and Conclusions

### 6.1. Summary

This paper presented for the first time a realistic simulation- and experimental measurement-based study on skull fracture detection with the MW technique. A comprehensive set of simulations was conducted with different head tissue layer models corresponding to different locations in the head, as well as using an anatomically realistic human head model. The measurements were performed with human tissue phantoms and a real human skull. Fourteen distinct study cases, utilizing S11/S21 evaluations and a power flow analysis, were presented to elucidate the impact of skull fractures. Two different antenna types were used in the simulations: a flexible antenna allowing the use of a wearable sensor and a directional cavity-backed antenna allowing also non-contact sensing. The evaluations were carried out with the flexible UWB antenna for the whole frequency band of 2–10 GHz to enable us to contemplate the impacts of skull fractures at different frequencies and hence assess the optimal frequency band for this application.

The results presented in this paper are promising: skull fractures even less than 1 mm could be detected easily with MW technique at different fracture locations. The power flow analysis gave insights into why the impact of the fractures may change with the frequency. The location and width of the fractures could be estimated both from the S11 and S21 results at the frequency ranges in which the changes are known to be the most visible with the selected antennas. Additionally, the S21 parameter could be used to estimate the length of the fracture. Detectability of the fractures could be further improved by reducing antennas’ coupling with the MDM approach.

### 6.2. Purpose and Novelty

The primary aim of this study was to evaluate the use of microwave techniques for detecting skull fractures and monitoring the healing process using realistic simulation and measurement models. Notably, this study is the first to model the healing process of skull fractures as connective tissue, providing the most realistic representation of the healing process to date. Additionally, this paper introduces novel assessments of the most promising frequency ranges for skull fracture detection within the ISM and UWB spectrum.

### 6.3. Advantages and Future Considerations for Portable Skull Fracture Detectors

Portable skull fracture detectors could be used as point-of-care diagnostics with several advantages. The main advantage is that skull fractures, their locations, and widths could be diagnosed already in ambulances or smaller healthcare centers. Especially, they would facilitate the diagnosis of children’s skull fractures faster. Portable skull fracture detectors would also allow frequent and safe monitoring of the fracture’s healing process. In particular, they would be beneficial for monitoring possible growing skull fractures, which can be challenging, especially in children [41]. Even though skull fractures seldom need surgery, they indicate that the force of the impact was substantial and the individual is at significantly greater risk of having an intracranial hemorrhage or brain injury [42]. Consequently, an observation in the hospital is required in most cases [43]. When comparing portable microwave-based devices to, for example, portable ultrasound devices, the former could serve as a continuous measurement method. This is due to the convenience of easily setting up antenna sensors embedded in a wearable device. The device could be used even for self-monitoring the healing process without nursing staff. Additionally, the MW-based technique also allows non-contact sensing, which could be a useful property, e.g., in monitoring the bone fracture healing process without the necessity of removing the casting.

This study provides insights into how microwave technology can be used to detect skull fractures in realistic models. Additionally, it demonstrates the critical importance of carefully selecting the frequency range, as detectability varies with frequency. However, before applying this method for practical implementation, comprehensive studies should be conducted for reference data determination. The reference data sets must take into account the natural variations in the dielectric properties and thickness of the human tissues. Additionally, this kind of application requires sensitive receivers with very low noise levels. Other topics for future work are stated in the Discussion section.

## Figures and Tables

**Table 1:** Dielectric properties of head tissue, retrieved from [21].

Tissue Layer	Relative Permittivity at 2, 4, 6, 8, 10 GHz	Conductivity [S/m] at 2, 4, 6, 8, 10 GHz	Wavelength [cm] 2, 4, 6, 8, 10 GHz
Skin	38.6; 36.6; 34.9; 33.2; 31.29	1.3; 2.3; 3.9; 5.8; 8.0	2.0; 1.0; 0.8; 0.6; 0.5
Fat	5.32; 5.12; 5.03; 4.94; 4.76; 4.6	0.09; 0.18; 0.31; 044; 0.58	6.4; 3.3; 2.2; 1.7; 1.4
Brain	36.7; 34.5; 32.4; 30.3; 28.4	1.0; 2.1; 3.7; 5.4; 7.3;	2.0; 1.0; 0.8; 0.7; 0.5
Cortical bone	11.6; 10.5; 9.6; 8.8; 8.1	0.3; 0.7; 1.2; 1.7; 2.1	4.3; 2.2; 1.6; 1.2; 1.0
Cancellous bone	19.1; 16.9; 15.2; 13.8; 12.6	0.65; 1.4; 2.2; 3.1; 3.9	3.3; 1.8; 1.3; 0.9; 0.8
Blood	59.0; 55.6; 52.2; 48.6; 45.1	2.2; 4.1; 6.8; 9.9; 13.13	1.9; 1.0; 0.7; 0.5; 0.4
Connective tissue	43.9; 40.2; 36.4; 32.7; 29.2	1.3; 3.2; 5.5; 7.9; 10.3	2.2; 1.1; 0.6; 0.4; 0.3
Difference between bone and blood	47.4; 45.1;42.6; 39.8; 37	1.9; 3.4; 5.6; 8.2; 11.0	
Difference between bone and connective tissue	32.2; 29.7; 26,8; 23.7; 21.2	1.0; 2.7; 3.2; 6.5; 8.9	

**Table 2:** Dimensions of the antennas.

Dimension	Antenna Type	
	Flexible Antenna (FA) (mm)	Directional Antenna (CA) (mm)
x	40	91
y	40	86
z	0.125	39.5
h1	11	25
h2	10	22.1
h3	9.1	3
w1	5.7	30.3
w2	3.8	3.35

**Table 3:** Power flow (Poynting vector) values at the selected points.

Frequency [GHz]	Model	Locations		
		A	B	C
5.5	Ref.	−66.3	−70.6	−77.1
	Blood	−68.1	−74.2	−82.2
	Connective	−66.7	−73.3	−80.7
9.0	Ref.	−80.1	−79.6	−81.2
	Blood	−81.6	−82.2	−85.5
	Connective	−83.5	−85.3	−87.6
9.8	Ref.	−79.5	−82.0	−79.5
	Blood	−79.0	−85.1	−86.2
	Connective	−78.9	−86.3	−86.4

**Table 4:** S11 and S21 parameters for different study cases.

Study Cases	Frequencies with the Best Detectability [GHz]	Maximum Differences	Study Cases	Frequencies with the Best Detectability [GHz]	Maximum Differences
**Case 1** (LM1, FA, HF, long)	S11 S21 4–4.6 (−) 3.8–4 (+) 5.4–5.6 (+) 4.8–5.2 (−) 8.5–9 (−) 8.5–10 (−)	S11 (5.5 GHz) S21 (9 GHz) 1 mm: 1.9 dB 1 dB 2 mm: 2.3 dB 2 dB 3 mm: 2.9 dB 2 dB 4 mm: 4.1 dB 2.5 dB 5 mm: 5.8 dB 3 dB	**Case 4** LM1, FA, vertical fracture	S11 S21 2.1 (+) 2.2–2.5 (−) 2.5–2.6 (−) 2.9–4 (+) 5.2–5.8 (+)	S11 (2.09 GHz) S21 (3 GHz) 1 mm: 8 dB 10 dB 2 mm: 10 dB 17 dB 3 mm: 12 dB 20 dB 4 mm: 14 dB 22 dB 5 mm: 16 dB 24 dB
**Case 1b** (LM1, FA, HF, short)	S11 S21 4–4.6 (−) 3–3.2 (−) 5.4–5.6 (+) 3.8 (−) 8.5–9 (−) 9.8 (−)	S11 (5.5 GHz) S21 (9 GHz) 1 mm: 1.9 dB 0.7 dB 2 mm: 2.3 dB 1.6 dB 3 mm: 2.9 dB 2 dB 4 mm: 4.1 dB 2.2 dB 5 mm: 5.8 dB 2.1 dB	**Case 5** LM1, CA, 23 mm	S11 S21 4.3 (−) 4.3 (−)	S11 (4.3 GHz) S21 (4.3 GHz) 1 mm: 1 dB 0.4 dB 2 mm: 2 dB 0.5 dB 3 mm: 2.5 dB 0.6 dB 4 mm: 3.5 dB 0.7 dB 5 mm: 4 dB 0.8 dB
**Case 1c** (LM2, FA, HF)	S11 S21 4.5 (+) 3–3.2 (−) 5.2–5.25 (+) 3.8 (−) 5.25–5.3 (−) 9.8 (−)	S11 (5.5 GHz) S21 (9 GHz) 1 mm: 0.5 dB 1 dB 2 mm: 1 dB 2 dB 3 mm: 2 dB 2 dB 4 mm: 2.5 dB 2.5 dB 5 mm: 3 dB 3 dB	**Case 5b** LM1, CA, 0 mm	S11 S21 3.7–4.1 (−) 3.8–4.2 (−) 4.1 (+)	S11 (4.3 GHz) S21 (3.72 GHz) 1 mm: 4 dB −0.8 dB 2 mm: 3.5 dB −0.85 dB 3 mm: 2.1 dB −0.9 dB 4 mm: 2 dB −1.1 5 mm: 1 dB −1.2
**Case 2** (LM1, FA, HF, connective tissue)	S11 S21 2.5–3 (+) 2.5–3 (−) 4–4.5 (−) 4–4.5 (+) 5.4–6 (−) 9.8 (−)	S11 (5 GHz) S21 (9.8 GHz) 1 mm: 1.5 dB 3.7 dB 2 mm: 2 dB 3.8 dB 3 mm: 3 dB 4.1 dB 4 mm: 3.5 dB 5 dB 5 mm: 4 dB 6 dB	**Case 6** Hugo, CA, 0 mm	S11 4.1 (−)	S11 2 mm: 1 dB 4 mm: 5 dB
**Case 3** Hugo head model, FA, long fracture	S11 S21 3 (+) 2–2.7 (+) 5.8 (−) 2.9–3.5 (+) 7.5 (−) 3.5–4.5 (−)	S11 (5.8 GHz) S21 (3 GHz) 2 mm: −14 dB −27.3 dB 4 mm: −12 dB −27.0 dB	**Case 7** Hugo, CA, 0 mm	S11 3.941 GH (+)	S11 2 mm 7.5 dB 4 mm 7.5 dB
**Case 3b** Hugo, FA, short fracture	S11 S21 3 (+) 2–2.7 (−) 5.8 (−) 2.9–3.5 (+)	S21 (3 GHz/4 GHz) 2 mm −6 dB/0.1 dB 4 mm +4 dB/0.2 dB	**Case 8** FA; skull	S11 2.5 GHz (−) 4–9 GHz (+)	S21 (4 GHz) 2 mm = 3.9 dB
**Case 3c** modified Hugo, FA, short fracture	S11 S21 2.2–3.8 (+/−) 2.5–4.5 (−) 6 (−) 7–8.5 (−) 9.5–10 (+)	S11 (6 GHz) S21 (3 GHz) 2 mm −7 dB 0.5 dB 4 mm −12 dB 1 dB	**Case 9** CA; skull	S11 2–3 (+) 3.5–4 (−)	S11 (3.8 GHz) −30 dB

**Figure A1:**
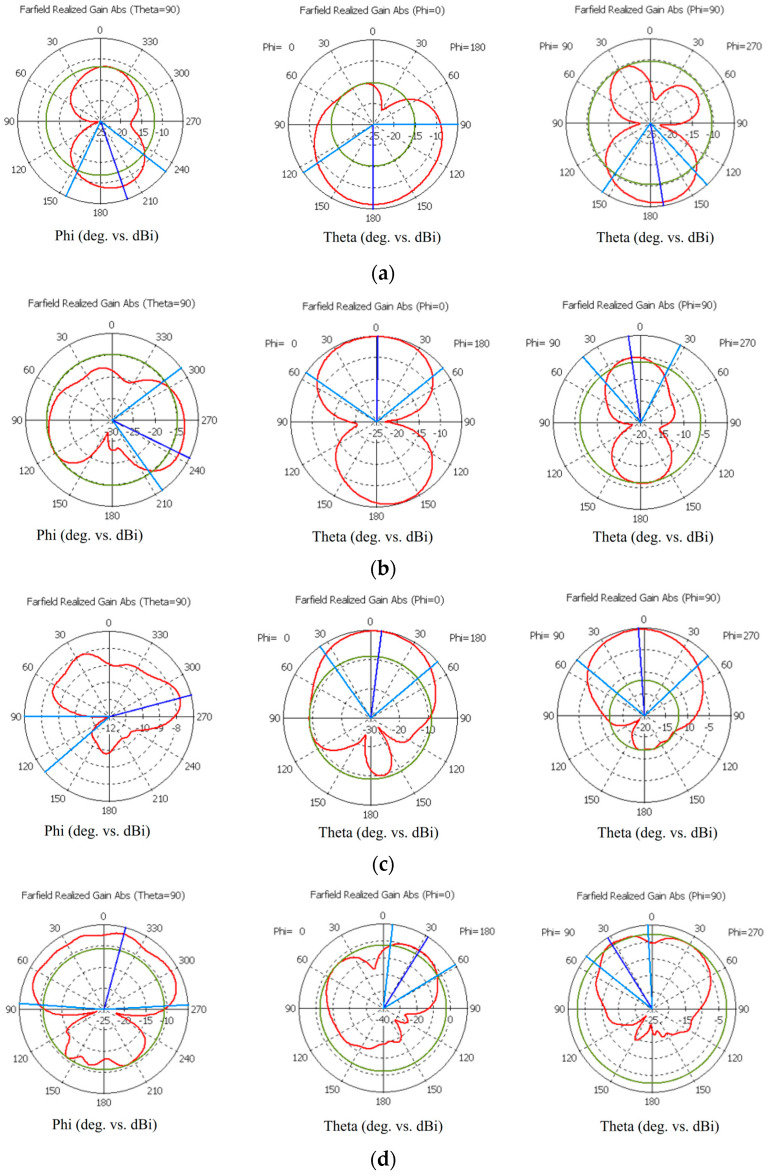
Simulated radiation characteristics (Theta = 90, Phi = 0, and Phi = 90) of the flexible antennas at frequencies (**a**) of the ISM band at 2.5 GHz, (**b**) 3.1 GHz, (**c**) 5.5 GHz, and (**d**) 9 GHz.

**Figure A2:**
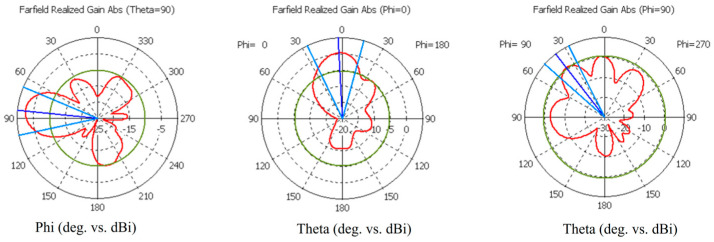
Simulated radiation characteristics (Theta = 90, Phi = 0, and Phi = 90) of the cavity-backed antenna at 4 GHz.

**Figure A3:**
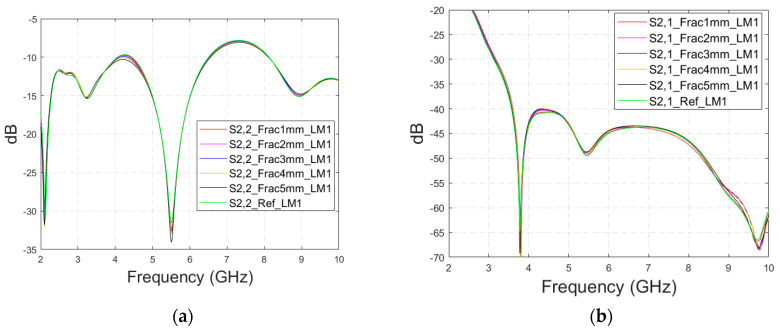

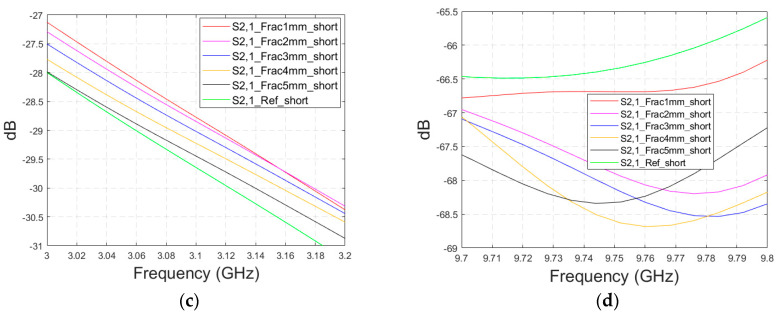
Simulated S22 and S21 results with layer model 1 with short horizontal fractures and flexible antennas: (**a**) S22 results with fracture widths of 1–5 mm for the whole simulated frequency range, (**b**) S21 results with fracture widths of 1–5 mm for the whole simulated frequency range, (**c**) S21 results zoomed to 3–3.2 GHz in which the differences are the largest, and (**d**) S21 results at 9.7–9.8 GHz.

**Figure A4:**
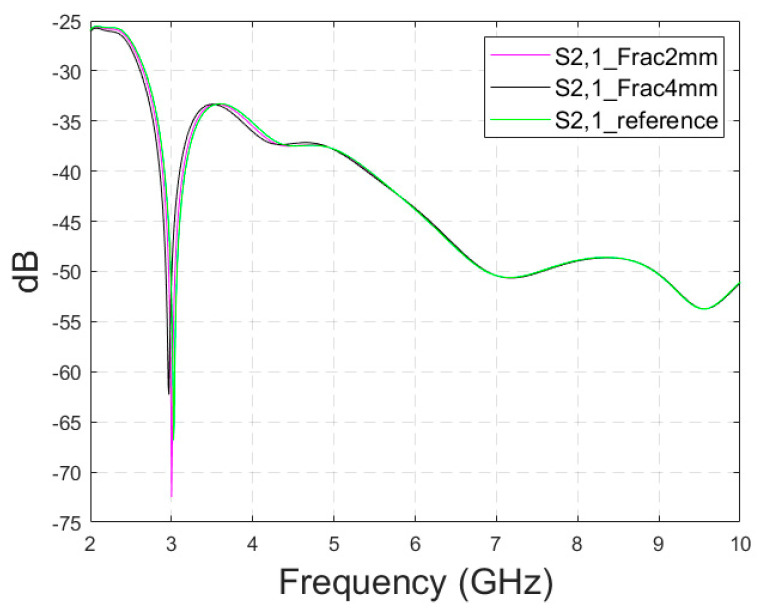
S21 parameters obtained using CST’s Hugo head model and flexible antennas for the reference case and shorter fractures having widths of 2 mm and 4 mm.

**Figure A5:**
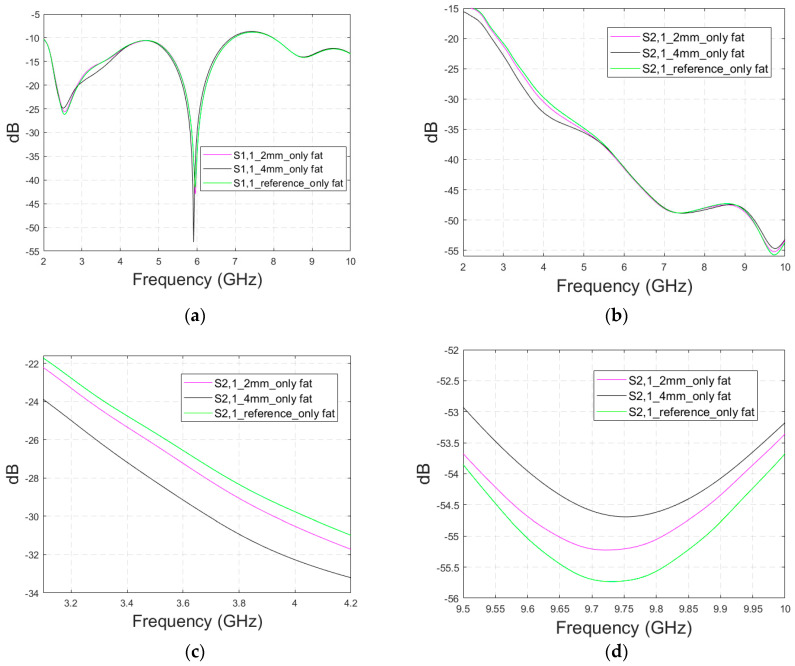
Simulated (**a**) S11 results and (**b**) S21 results for the Hugo head simulation model having averaged muscle–fat tissue replaced with pure fat tissue for the range of 2–10 GHz, (**c**) the zoomed S21 at 3.15–4.2 GHz, and (**d**) the zoomed S21 at 9.5–10 GHz.

**Figure A6:**
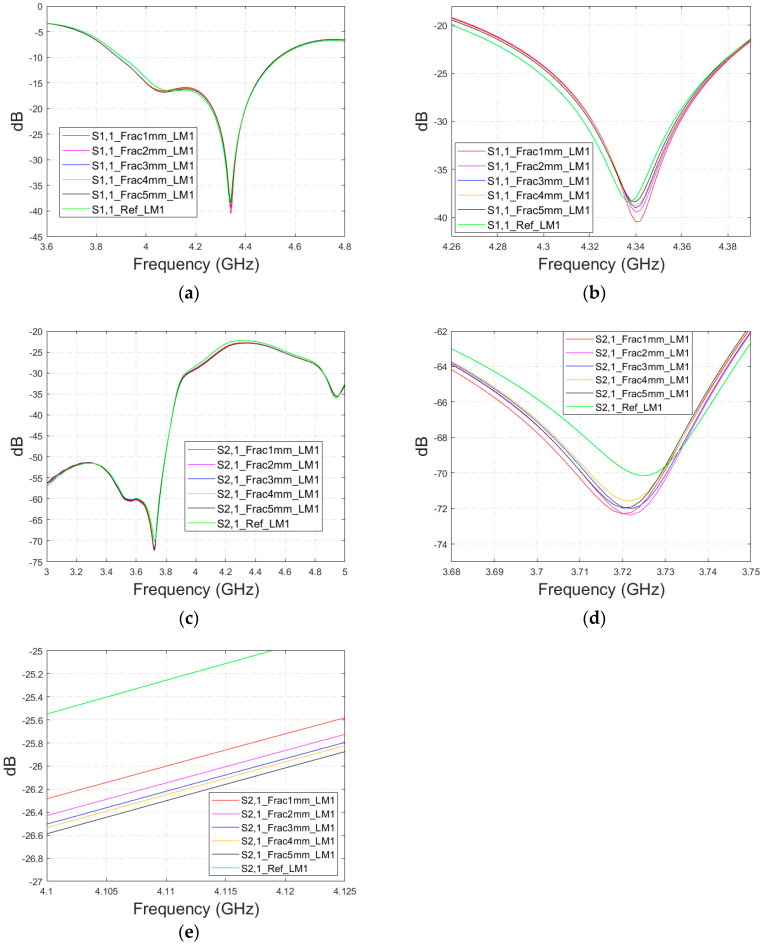
Simulated S11 and S21 results obtained using cavity-backed antennas with a 0 mm antenna–skin distance and layer model 1 with horizontal fractures filled with blood: (**a**) S11 results with fracture widths of 1–5 mm for the whole simulated frequency range and (**b**) zoomed to 4.31–4.37 GHz in which the differences are the largest, (**c**) S21 results for the whole frequency range, (**d**) S21 at 3.68–3.75 GHz in which the differences are at largest, and (**e**) S21 at 4.1–4.12 GHz in which the fracture effects gradually decrease with the S21 parameter.

## Data Availability

The original contributions presented in the study are included in the article, and further inquiries can be directed to the corresponding author.

## Appendix A

Appendix presents additional evaluation results for skull fracture detection studies carried out with CST simulations. The following cases are investigated:

- Radiation patterns for on-body antennas used in the evaluations;
- Case 1c—layer model 1 with a shorter fracture length;
- Case 3b—realistic simulation model with a shorter fracture;
- Case 3c—modified head simulation model with a pure fat layer;
- Case 5b—layer model 1, horizontal fracture, cavity-backed antenna with a distance of 0 mm.
- The results are included in Table 3, which summarizes all the studied cases.

### Appendix A.1. Radiation Pattern for On-Body Antennas

This subsection presents the radiation characteristics of the on-body antennas used in the evaluations. The spherical coordinates are presented in Figure A1a. Polar plots, including *Theta = 90, Phi = 0 and Phi = 90* plots, are presented for the flexible antennas shown in Figure A1b–d for the frequencies of 2.5 GHz, 3 GHz, 5.5 GHz, and 9 GHz. Additionally, polar plots of the cavity-backed antenna are presented in Figure A2. As it can be seen, both antennas have strongest lobes outwards in the layer model, which is natural with on-body antennas, even despite the cavity. There are also notchy areas in the patterns towards the layer model, which have clear impacts on how the signal propagates inside the tissue.

### Appendix A.2. Case 1c: Layer Model 1 with a Shorter Fracture Length

This subsection presents the evaluations for the impacts of shorter fractures with the LM1 simulation model. The fracture has a length of 4.1 cm, reaching only the middle part of the model (fracture located only below antenna 2). In this case, the results for the S22 parameter are presented in the fracture below antenna 2. The S22 and S21 results are shown in Figure A3a–b for the whole frequency ranges, respectively, and S21 results zoomed in Figure A3c for 3–3.2 GHz and in Figure A3d for 9.7–9.8 GHz. As it can be seen, the fracture also causes clear changes in the S21 parameter in the case of shorter fractures. Naturally, the differences are slightly smaller than in Case 1, though they are still clearly visible. These results further confirm the idea that it is essential to analyze the S21 parameter in addition to the S11/S22 parameters, since the S21 value may provide information on the length and location of the fracture.

### Appendix A.3. Case 3b: Realistic Simulation Model with a Shorter Fracture

Next, the impacts of shorter fractures are evaluated with the Hugo head model. Fractures with widths of 2 mm and 4 mm have a length of 4.1 cm, reaching only the middle part of the head model (fracture only below the other antenna), as in Case 4 with the layer model. S21 results for the whole simulated frequency range are shown in Figure A4. In this case, the differences are visible in the same frequency range as with longer fractures, 2–4.5 GHz, but the differences are smaller, as expected. For instance, in the notch area, the differences between S21 of the reference and fracture cases are −5 dB and +6 dB. Although the differences are clear, the challenge is that they are mostly visible only in the notch area. Additionally, it may be confusing that the 2 mm fracture decreases attenuation, whereas the 4 mm fracture increases attenuation. At 4 GHz, differences between the fracture and reference cases are very small, around 0.1–0.2 dB. Moreover, no differences can be seen at higher frequencies. The main reason for this is that the Hugo head simulation model has a 10 mm thick average fat and muscle layer between the skin and skull (based on the authors’ observation from the simulation model). In a real human head, there is no muscle layer on the top of the head in which the simulations are carried out. Additionally, the fat layer thickness on top of the head is only 1.4 mm [22]. Since the propagation loss in the muscle layer is significantly higher than in the fat layer, the fractures are more challenging to detect with the simulation model having an averaged muscle–fat layer, especially if it is significantly thicker than in realistic cases. Similarly, the differences between the reference and fracture cases are found to be smaller with layer model 2, which includes separate muscle and fat layers.

### Appendix A.4. Case 3c: Modified Head Simulation Model with a Pure Fat Layer

In this subsection, the impact of short fractures is evaluated by a modified head simulation model in which the averaged muscle–fat tissue between the skull and the skin is replaced with pure fat tissue to correspond a more realistic scenario on the top of the head. However, the thickness of the model cannot be modified and hence the model has still approximately 7 times thicker fat tissue on the top of the head than in the average scenario.

The S11 and S21 results are shown for the whole simulated frequency range in Figure A5a,b, respectively. As expected, the changes between the fracture and reference cases are found to be slightly larger since the propagation loss in pure fat tissue is clearly lower than with the averaged muscle–fat tissue. In the S11 parameter, shown in Figure A5a, the clearest changes can be seen at 2.5–3.5 GHz and 5.9 GHz. In the S21 parameter, which is presented for the whole frequency range in Figure A5b, the clearest changes can be seen around 3.5 GHz, as depicted in Figure A5c. Interestingly, S21 is smoother than in Case 3a. For instance, the notch in the S21 parameter disappears when pure fat tissue is used. The differences between reference and fracture cases can be noted also at higher frequencies, e.g., at 9–10 GHz, as seen in Figure A5d which was not possible in Case 3.

In general, the results obtained with the anatomical head simulation model in Cases 5–7 are promising: despite the significantly thicker tissue layer between skin and bone, some detectable differences can be seen in both in S11 and S21. In more realistic scenarios, in which the tissue layer between the skin and skull would be thinner, the changes are expected to be remarkably clearer. Another aspect that may affect the simulation results is that the head simulation voxel model has a non-smooth shape and hence there are some unintentional air gaps between the antennas and skin layer. Since the flexible antennas are designed to operate with a minimal antenna–skin distance, the additional air between the antenna and skin tissue may influence the antenna characteristics negatively and hence deteriorate detectability. However, in realistic situations, as the flexible antenna can be attached directly to the skin to follow the skin surface smoothly, this problem is assumed to be negligible.

### Appendix A.5. Case 5b Layer Model 1, Horizontal Fracture, Cavity-Backed Antenna with a Distance of 0 mm

In this subsection, an antenna–skin distance of 0 mm is evaluated with the cavity-backed antenna. This antenna distance is not optimal for the cavity-backed antenna’s operation since it is designed to operate best with a 20–30 mm antenna–body distance. The S11 results are presented in Figure A6a,b for the simulated frequency range of 3–5 GHz and the zoomed version at 4.31–4.37 GHz, where the differences are the most visible. The differences between the reference and fracture cases are easily detectable: the largest S11 difference is at 4.31 GHz, as the 1–5 mm width fracture causes a 1–4 dB reduction in S11 parameter. However, the order of reduction is illogical in this case: an 1 mm fracture causes 4 dB reduction in the S11 parameter and a 4 mm fracture causes a 1 dB reduction.

In the S21 results, which are presented in Figure A6c–e, the differences are smaller in general but visible throughout the whole frequency bandwidth. The largest difference between the reference and fracture cases can be seen at 3.72 GHz, in which the difference is 2.6 dB, as shown in Figure A6d. Moreover, in this case, the impact of the fracture does not increase/decrease logically since the largest difference appears with a 1 mm fracture and the smallest with a 4 mm fracture. However, at 4 GHz, the S21 differences due to fractures are smaller (0.8–1.2 dB) but the changes are logical: the S21 parameter gradually decreases as the fracture width increases.

In general, the differences in S parameters caused by fractures are smaller in Case 5b than in Case 5, in which the antenna–skin distance was 23 mm. However, the results for Case 5b prove that the fractures can also be detected with the directional antenna with smaller antenna–skin distances.
